# Environmentally Acquired *Bacillus* and Their Role in *C. difficile* Colonization Resistance

**DOI:** 10.3390/biomedicines10050930

**Published:** 2022-04-19

**Authors:** William T. Ferreira, Huynh A. Hong, James R. G. Adams, Mateusz Hess, Natalia K. Kotowicz, Sisareuth Tan, Enrico Ferrari, Alain Brisson, Jurgen Zentek, Mikhail Soloviev, Simon M. Cutting

**Affiliations:** 1 Department of Biological Sciences, Royal Holloway University of London, Egham TW20 0EX, UK; wuaz082@live.rhul.ac.uk (W.T.F.); hong.huynh@rhul.ac.uk (H.A.H.); ja01329@surrey.ac.uk (J.R.G.A.); mateusz.hess@kcl.ac.uk (M.H.); 2 SporeGen Ltd., London Bioscience Innovation Centre, 2 Royal College Street, London NW1 0NH, UK; natalia.1.kotowicz@kcl.ac.uk; 3 Laboratoire d’Imagerie Moléculaire et Nano-Bio-Technologie, UMR-CBMN CNRS-Université de Bordeaux-IPB, 33607 Pessac, France; s.tan@u-bordeaux.fr (S.T.); alain.brisson@u-bordeaux.fr (A.B.); 4 School of Life Sciences, University of Lincoln, Lincoln LN6 7TS, UK; eferrari@lincoln.ac.uk; 5 Institute for Animal Health, Freie University of Berlin, Berlin 14195, Germany; zentek.juergen@vetmed.fu-berlin.de

**Keywords:** *Clostridioides difficile* infection, *Bacillus*, environmental bacteria, allochthonous bacteria, lipopeptides

## Abstract

*Clostridioides difficile* is an environmentally acquired, anaerobic, spore-forming bacterium which ordinarily causes disease following antibiotic-mediated dysbiosis of the intestinal microbiota. Although much is understood regarding the life cycle of *C. difficile*, the fate of *C. difficile* spores upon ingestion remains unclear, and the underlying factors that predispose an individual to colonization and subsequent development of *C. difficile* infection (CDI) are not fully understood. Here, we show that *Bacillus*, a ubiquitous and environmentally acquired, spore-forming bacterium is associated with colonization resistance to *C. difficile*. Using animal models, we first provide evidence that animals housed under conditions that mimic reduced environmental exposure have an increased susceptibility to CDI, correlating with a loss in *Bacillus*. Lipopeptide micelles (~10 nm) produced by some *Bacilli* isolated from the gastro-intestinal (GI)-tract and shown to have potent inhibitory activity to *C. difficile* have recently been reported. We show here that these micelles, that we refer to as heterogenous lipopeptide lytic micelles (HELMs), act synergistically with components present in the small intestine to augment inhibitory activity against *C. difficile*. Finally, we show that provision of HELM-producing *Bacillus* to microbiota-depleted animals suppresses *C. difficile* colonization thereby demonstrating the significant role played by *Bacillus* in colonization resistance. In the wider context, our study further demonstrates the importance of environmental microbes on susceptibility to pathogen colonization.

## 1. Introduction

Despite substantial progress in our understanding of *Clostridioides difficile* infection (CDI) [1], several questions remain. Notably, why an increase in cases has arisen over the past 30 years [2], how *C. difficile* is able to circulate amongst the healthy population, including infants [3], and what factors are involved in preventing *C*. *difficile* colonization. With regards to the latter question, advances in treatment, such as fecal microbiota transplantation (FMT), have shown that soluble compounds within fecal material are able to inhibit *C. difficile*, suggesting an extracellular contribution to resistance [4,5]. In humans, *C. difficile* is acquired by the majority of infants during the first six months after birth [6,7]. After about one year the intestinal population of *C. difficile* begins to decline and by the end of the second is mostly absent [8]. To account for this reduction, the acquisition of a mature, adult microbiota is thought to install a barrier to colonization [8]. However, it has been shown that the diversity of an infant’s microbiota often does not reach that of adults until the 4th year [9], suggesting that other more specific changes may be responsible for the decline in *C. difficile*. One significant change is the dietary acquisition of microbes critical for short chain fatty acid (SCFA) production and the formation of secondary bile acids [10], with members of the *Clostridium* genus being identified as important contributors with regards to the latter [11,12].

Animals, sharing close physiological relatedness to humans, exhibit a similar pattern of early *C. difficile* colonization with, in pigs for example, all (100%) animals being asymptomatically colonized within two days of birth [13]. However, unlike in humans, *C. difficile* persists for, at most, two months following birth [14,15]. The lifestyle of pigs and humans is clearly different regarding diet and nutrition, but also with regard to the environs to which piglets are immediately exposed, while for infants this is often delayed until at least one year of age. This raises the question: what impact and by which mechanisms can the acquisition of environmental microbes influence colonization resistance? The acquisition of bacteria from the environment, including aerobic spore formers, has been linked to a decreased presence of pathogens in the GI-tract; pigs housed in a natural outdoor environment show an increased prevalence of Firmicutes and a decreased abundance of pathogenic populations, while animals raised in more hygienic indoor environments exhibit the opposite trend [16]. Indeed, this was exemplified in a recent study demonstrating that the administration of *Bacillus* to pigs whose gut flora had been depleted by antibiotics cured them of diarrhea [17]. Incidentally, passive processes (hygiene, lifestyle and diet) together with the acquisition of environmental bacteria have all been implicated as factors impacting upon the human gut microbiota and the prevention of pathogen colonization [18].

The *Bacilli* form robust, gastric-resistant, endospores commonly found in soil, air and foods which can transiently proliferate in the mammalian GI-tract [19]. *Bacilli* are able to produce a large and diverse array of ribosomal and non-ribosomal antimicrobials [20] under a variety of environmental conditions. These structurally diverse antimicrobial compounds suppress the growth of competing bacteria and pathogens, and the production of these compounds have been shown to increase in the presence of competitors [20,21,22]. *Bacillus*-produced antimicrobial peptides have been shown to be inhibitory against numerous pathogens, including *Clostridium perfingens, Escherichia coli, Listeria monocytogenes* and *Salmonella* spp. [22]. The targets of these antimicrobials are typically microbes that have coevolved in a shared environmental niche with *Bacillus*. Contact between different coevolved bacterial species in new environs can elicit phenotypic changes or the modulated production of antimicrobial peptides [20]. Included amongst these antimicrobials are amphipathic lipopeptides which, at high concentrations, form micelles (~6–10 nm) able to entrap other antibiotics produced by the host bacterium [23]. These micellar antibiotics, which we refer to as HELMs (heterogenous lipopeptide lytic micelles), are able to associate with the surface of the cell or spore, further enhancing targeting and activity [23]. While many types of bacteria are found in the environment, the inherent robustness and ubiquity of spore formers, such as *Bacillus,* makes them ideally adapted for acquisition by an animal host. Many studies have relied upon 16S rDNA metagenomic sequencing to identify changes in the composition of intestinal microbiota found in the host [24,25]. These methods have proven useful in understanding gut dysbiosis, however, they are unable to resolve between species. In the case of the endospore forming Firmicutes, under-detection and under-reporting have presented a recurrent issue in metagenomic datasets [26].

Indeed, in a recent study, environmentally acquired *Bacillus* were shown, using culture-based methodology, to play an integral role in colonization resistance to *Staphylococcus aureus* in the human GI-tract [27]. Interestingly, a correlation between the presence of *B. subtilis* and the absence of *S. aureus* was only found when using direct culture methods, with 16S rRNA metagenomic sequencing showing no association between the two species. Fermented foods, later found to be enriched with *Bacillus* spores, have been used medicinally for centuries [28], and yet a role for *Bacillus* in CDI has not previously been noted. Here, we report that the presence of *Bacillus* spp. is reduced following diminished host environmental exposure or the administration of antibiotics. Additionally, the increased presence of *Bacillus* spp. was associated with a decreased presence of *C. difficile* in the GI-tract. *Bacillus* spp. were found to produce potent antimicrobials which act upon *C. difficile* in vitro and in vivo, and the restoration of physiological levels of *Bacillus* to an antibiotic-depleted host-restored colonization resistance to *C. difficile*.

## 2. Materials and Methods

### 2.1. General Methods and Strains

General methods and media for work with *Bacillus* were as described [29]. The growth media used for *Bacillus* was either LB, BHIB (Brain Heart Infusion Broth) or DSM (Difco Sporulation Medium). For *C. difficile,* the culture was made anaerobically using a Don Whitley chamber using Brain heart infusion with (BHISS) or without (BHIS) sodium taurocholate (0.1% *w*/*v*) [30]. ChromID (BioMerieux) is a selective medium containing cefoxitin and cycloserine and used for *C. difficile* plate culture. Spores were prepared on SMC agar and further purified using centrifugation through a 20% (*v*/*v*) to 50% (*v*/*v*) Histodenz gradient (Sigma), as described [30]. The *C. difficile* laboratory reference strain CD630 (ribotype 012, *tcdA^+^ tcdB^+^*) was used for in vitro, ex vivo and in vivo studies. The aims and methodologies in this study are described schematically (Supplementary Figure S1).

### 2.2. Analysis of Bacillus and C. difficile Spores in Fecal Samples

For routine analysis of spores in fecal samples (e.g., from neonatal piglets, mice or hamsters), samples were homogenized in dH_2_O and heat-treated (65 °C for 45 min.) and dilutions plated on selective ChromID medium (*C. difficile*) or LB (*Bacillus*) with anaerobic and aerobic culture (37 °C, 48 h), respectively. Human-derived samples had been collected in previous work [31]. The identity of *C. difficile* colonies was confirmed by selecting three representative colonies and performing colony PCR targeting 16S rRNA using CD630 as a positive control [32]. The identity of representative *Bacillus* colonies was confirmed by analysis of the *gyrA* DNA sequence, as previously described [33,34].

### 2.3. Screening of Spore Formers in Fecal Samples with Activity Against C. difficile

Pooled fecal samples taken from mice or hamsters before and after (24 h) clindamycin treatment (i.g.; 30 mg/kg) were used to identify heat-resistant aerobic bacterial colonies. Colonies (minimum of 500) were re-streaked and overnight cultures (2 mL) made in LB broth. The cell-free supernatant was filter-sterilized (0.45 μm pore size) and assessed for inhibitory activity to CD630 using an in vitro microdilution assay [23]. Plates were set up as follows: 216 µL of BHIS growth medium was pipetted into the first row of a 96-well U-bottom microplate (Greiner Bio-One, Gloucestershire, UK) and followed by 120 µL into each subsequent row. Next, 24 µL of the supernatant to be tested was pipetted into the first row (1:10 dilution factor) and serially diluted in a 2-fold dilution series until the last row (1:1280 dilution factor) on the microplate. A media-only control was also applied into a single well of the first column and serially diluted. Subsequently, 12 µL of a 6 h CD630 culture was added to each well and the plate was incubated overnight at 37 °C. The following day, the plate was agitated on a rotary plate shaker (200 rpm, 2 min.) after which the OD_600_ was read using a microplate reader. Positive inhibitory activity was defined as an OD_600_ < 50% of the CD630 media-only control. For non-sterile samples, ‘CD supplement’ (Sigma-Aldrich) was added to the BHIS. Preliminary species assignment was made by analysis of the *gyrA* DNA sequence [33,34]. In all cases, active isolates were bacteria whose culture supernatants showed inhibition at a dilution of >1/10 in an in vitro microdilution assay.

### 2.4. Purification and Identification of Heterogenous Lipopeptide Lytic Micelles (HELMs) from Bacillus Bacterial Culture

Purification of HELMs from culture including ammonium sulphate (AmSO_4_) precipitation, size exclusion chromatography (SEC), RP-HPLC, dynamic light scattering (DLS) and matrix-assisted laser desorption ionization time-of-flight mass spectrometry (MALDI-TOF-MS) analyses have been described previously [22]. Briefly, HELMs were precipitated overnight at 4 °C in 20% (*w*/*v*) of AmSO_4_ from filter-sterilized, cell-free culture supernatant (0.45 µm). The resulting precipitate was resuspended in and dialyzed against sterile PBS, followed by fractionation using SEC with Superdex 200 resin and PBS as a running buffer. The active SEC fraction was further fractionated by RP-HPLC using a uBondaPack Phenyl 125 Å, 10 µm, 3.9 × 300 mm column and Waters 600 Multisolvent Delivery system. The compounds were separated using a linear gradient of 60% to 95% (*v*/*v*) methanol in 0.5% (*v*/*v*) acetic acid at a flow rate of 0.5 mL/min, absorbance was monitored at 220 nm. Individual fractions were concentrated using an EZ-2 Genevac centrifugal evaporator and identified using Bruker Autoflex III MALDI-TOF-MS. The active compounds were confirmed at each step of purification by testing against CD630 in a microdilution assay.

### 2.5. Synergy between RP-HPLC Separated Lipopeptides

The SEC ‘active’ sample was separated by RP-HPLC and identified by MALDI-TOF, as described [23]. To assess levels of activity and synergy between individual components, specific fractions were combined and then vacuum evaporated. Fractions were resuspended in dH_2_O and tested for activity against CD630 using the microdilution assay.

### 2.6. HELM Bacteriolysis of C. difficile in Culture

Experiments were conducted using mid-logarithmically growing cultures of CD630 (6 h), dividing the culture into four and adding 1:10 volume of a cell-free supernatant of Bv277 (HELM^+^) or *srfAA*- (HELM^−^). The HELM^+^ (Bv277) SEC-fractionated ‘active’ fraction was used at a dilution factor of 1:100 (*v*/*v*) and PBS was used as a negative control. OD_600_ measurements were taken hourly. Aliquots were taken hourly, serially diluted in PBS and plated onto ChromID agar to enumerate the viable CFU.

### 2.7. DLS Analysis

The Bv277 supernatant was AmSO_4_ precipitated and then separated by SEC. The SEC fraction was vacuum evaporated and dry material was resuspended in sodium phosphate buffer (150 mM, pH 7.2) and cleared by centrifugation (17,000× *g*, 1 h). For analysis, 100 µL was transferred to a micro-cuvette, equilibrated at 25 °C and measured in triplicate using a Zetasizer Nano ZS (Malvern, Australia). The diameter was estimated from the Z-average size using Zetasizer software v7.11 (Malvern, Australia).

### 2.8. RP-HPLC and MALDI-TOF Identification of HELMs in Small Intestinal (SI) Samples

Groups of mice (n = 3) were treated as follows: (i) untreated mice, (ii) treated with 30 mg/kg clindamycin. A total of 24 h after the study commenced the SI contents were removed, homogenized in 1 mL dH_2_O, shaken vigorously for 1 h at RT and then transferred into 9 mL 100% MeOH for 3 h. The extracts were cleared by centrifugation at 12,000× *g* for 20 min and evaporated under vacuum. The dried material was resuspended 1:3 in PBS (*w*/*v*) according to the original weight. The MeOH-extracted SI contents in PBS were diluted with 100% MeOH to a final concentration of 20% and a final volume of 5 mL. The diluted extracts were centrifuged at 21,000× *g* for 10 min. and the supernatants were removed. Centrifugation was repeated twice under the same conditions to ensure removal of all particulates. All buffers used with Sep-Pak C18 cartridges (Waters) had acetic acid added to a final concentration of 0.1% (*v*/*v*). Sep-Pak C18 cartridges were activated with 10 mL of 100% MeOH before being equilibrated with 10 mL of 20% (*v*/*v*) MeOH. The supernatants were then applied to the column, the flow through collected and reapplied to the same cartridge again (twice more). The columns were then washed with 10 mL of 20% (*v*/*v*) MeOH and elution was performed with 2 mL of 100% MeOH. The eluted fractions were evaporated for 3 h using a vacuum evaporator. Dried samples were resuspended at a 10:1 (*w*/*v*) ratio in 60% (*v*/*v*) MeOH according to the original weight of the intestinal contents. Samples were then analyzed using RP-HPLC by injecting 50 μL (representing 0.5 g intestinal contents), and lipopeptides were detected using MALDI-TOF-MS, as described above and elsewhere [22].

### 2.9. Methanol (MeOH) Extraction of Intestinal Contents

Groups of mice (n = 3) were treated as follows: (i) untreated mice, (ii) treated with 30 mg/kg clindamycin, (iii) treated with 30 mg/kg clindamycin and after 20 h dosed orally with Bv277 (HELM^+^) or (iv) *srfAA*^−^ (HELM^−^) spores (~2 × 10^9^ CFU). A total of 27 h after the study commenced the SI contents were removed, homogenized in 1 mL dH_2_O, shaken vigorously for 1 h at RT and then transferred into 9 mL 100% MeOH for 3 h. The extracts were cleared by centrifugation at 12,000 *g* for 20 min. and evaporated under vacuum (Genevac EZ-2). The dried material was resuspended 1:3 in PBS (*w*/*v*) according to the original weight. Samples were kept at 4 °C overnight to allow micellar assembly of amphiphilic components before experimentation. These SI extracts were then used for ex vivo analysis of inhibitory activity against *C. difficile* and the SI synergy experiment.

### 2.10. Ex Vivo Analysis of Inhibitory Activity Against C. difficile

The MeOH-extracted SI contents in PBS (1:3, *w*/*v*) were diluted 1:1 in BHIS (+2% (*w*/*v*) sodium taurocholate), combined immediately with CD630 spores (1 × 10^6^ CFU) and incubated anaerobically at 37 °C. PBS was used as a control in place of SI extracts. Samples were removed for analysis of total counts of CD630 CFU by plating on selective ChromID agar. Additionally, heat resistant spores (60 °C, 30 min.) were enumerated on ChromID agar and all samples at 1 h showed no counts and therefore complete germination of all CD630 spores.

### 2.11. Synergy between HELMs and SI Extracts

A sterile, cell-free Bv277 supernatant was incubated for 3 h (RT) 1:1 with: dH_2_O (HELMs) or methanol extracts of mouse SI with (HELMs + SI-Clin) or without (HELMs + SI) clindamycin treatment. Methanol extracts of mouse SI had been evaporated and resuspended in saline 1/3 (*w*/*v*) according to the original weight of the intestinal contents. The mixtures were serially diluted and inhibitory activity to CD630 was measured using the microdilution assay.

### 2.12. Synergy between HELMs and DOC

Varying concentrations of Bv277 HELMs (1 to 4 μg/mL) were mixed with different concentrations of primary bile acid cholate (CA) or secondary bile acid deoxycholate (DOC) (50 to 400 μM) and added to mid-logarithmic cultures of CD630 and incubated for 5 h at 37 °C. OD readings (600 nm) were taken, and relative growth determined as the percentage increase in OD_600_ from the time of addition of samples to the CD630 culture. Concentration of lipopeptides was estimated according to the dry weight of purified SEC fractions.

### 2.13. In Vivo Studies

C57 BL/6 mice (females, aged 10–12 weeks, Charles River) were used for all murine studies. Golden Syrian Hamsters (females, aged 16–18 weeks old; Envigo) were used for the hamster study. All animal procedures were performed under the UK Home Office project license PPL 70/8276. For enumeration of *C. difficile* spores in feces or cecum, samples were homogenized in 70% ethanol, incubated overnight, serially diluted in sterile water and plated onto selective ChromID. Plates were incubated anaerobically for 2 days (37 °C) before counting [35]. Toxins A and B in fecal and cecal samples were quantified using ELISA (Supplementary Methods) [35].

### 2.14. Preparation of Bacillus Test Material for In Vivo Studies

All *Bacillus*-derived test material used for the dosing of animals was grown in BHIB from a single colony for 8 h (200 rpm, 37 °C) prior to being sub-cultured at a dilution of 1/100 in 100 mL of BHIB and incubated for 16–18 h (200 rpm, 37 °C). The material was centrifuged (10,000× *g*, 10 min.), the supernatant retained and the pellet washed two times and resuspended in PBS, serially diluted and counted on DSM plates to determine the CFU/mL of the original culture. The resultant material consisted of >95% spores (data not shown). The cell-free supernatant was filter sterilized (0.45 μm pore size) and stored at −20 °C.

### 2.15. Murine C. difficile Colonization Susceptibility Study

Mice receiving non-sterilized food, water, bedding and exposed to a non-filtered environment are referred to as ‘Conventionally caged’ (CC). Mice receiving UV-treated food, water and bedding and exposed to a HEPA-filtered IVC environment are referred to here as ‘Super Clean’. Mice (n = 5/gp) were housed in CCs or IVCs (Super Clean) for 12 months to mimic the increased susceptibility observed in elderly humans [36] after which they were administered clindamycin (i.g.) at different doses, followed 24 h later by challenge (i.g.) with 10^2^ spores of CD630. Animals were considered colonized when carrying detectable levels (>10^3^/g CFU) of ethanol-resistant *C. difficile* spores (ChromID) in their ceca 24 h post challenge [35]. The infectious concentration 50% (IC_50_) of the clindamycin was determined in mice using Graphpad Prism software (v. 9.1.2, Graphpad Software Inc., San Diego, CA, USA). Counts of *Bacillus* spores from individual mice were collected 24 h prior to clindamycin administration and enumerated as described above.

### 2.16. Bacillus and C. difficile Colonization Cohort Study in Piglets

Fecal samples were collected from neonatal piglets (n = 3) housed in pens at 1, 2, 3, 5, 7, 10 and 13 days post-birth. Each collected fecal sample was analyzed for both *C. difficile* spores (ChromID) and aerobic *Bacillus* (LB) spores, as described above.

### 2.17. Hamster Clindamycin Colonization Model

Hamsters were housed individually in IVCs with 6 animals/gp. The study initiated with a single oral (i.g.) dose of clindamycin (30 mg/kg). Groups were then dosed two times (2 × 10^9^ CFU/dose) with either HELM^+^ or HELM^−^ *Bacillus* spores or HELM^+^ cell-free supernatant (2 mL/dose). A total of 24 h post-clindamycin the animals were given a single oral (i.g.) dose of CD630 pure spores (10^2^ CFU), after which they received a further three doses of *Bacillus* spores or supernatant. Dosing of *Bacillus* spores or supernatant (3-times/day, 6 h intervals) continued till study end. Animals were monitored for symptoms of disease progression and culled upon reaching the clinical endpoint. Symptoms of CDI scored were defined as severe/clinical end point (wet tail >2 cm, high lethargy), mild (wet tail <2 cm) or healthy. Ceca were taken from culled animals and analyzed for the presence of toxins by ELISA and ethanol resistant *C. difficile* spore CFU (ChromID), as described above.

### 2.18. Murine Microbiota Depletion Model

Mice were housed in groups (n = 6/gp) in IVCs and for 7 days were given an antibiotic cocktail (1 g/L of vancomycin, kanamycin, ampicillin, metronidazole, gentamicin and 30 g/L of sucrose) ad libitum in their drinking water to eradicate the pre-existing intestinal microbiota. The antibiotic cocktail was replaced every 2 days with fresh antibiotic and after 7 days mice were given ordinary drinking water for 2 days. During this washout period mice were dosed three times per day with either 10^7^, 10^6^, 10^5^, 10^4^, 10^3^ CFU/dose of HELM^+^ or HELM^−^ *Bacillus* spores or PBS (naïve group). On the day of challenge, mice received a further three doses of *Bacillus* spores and were also challenged with 10^3^ CD630 spores. A total of 24 h after challenge mice were culled and the cecum removed to determine ethanol-resistant *C. difficile* spore counts (ChromID) and levels of toxin A, as described above. Feces from each mouse were collected, serially diluted in PBS and plated on LB or BHIS under aerobic or anaerobic conditions, respectively, after antibiotic treatment (37 °C, 48 h) to ensure that the microbiota had been depleted of bacteria (data not shown).

### 2.19. Statistics

**Figure 1:**
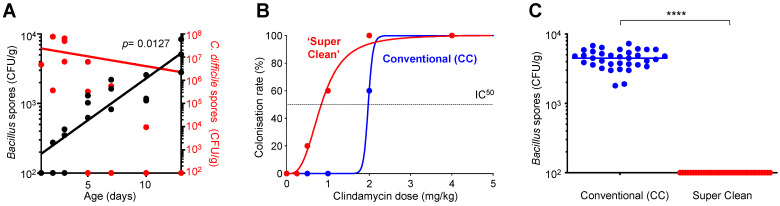
*C. difficile* colonization is inversely correlated with the acquisition of environmental *Bacillus*. (**A**) *C. difficile* colonization in piglets decreased following environmental exposure after birth. Fecal samples of neonatal piglets were collected during days 1–13 following birth. *C. difficile* (red circles) and *Bacillus* spores (black circles) were enumerated from the same fecal sample, from each individual piglet. A statistically significant negative correlation (Spearman’s rank-order) was observed between counts of *C. difficile* and *Bacillus* spores in pig fecal samples (r_s_ = −0.5464, *p =* 0.0127). A significant correlation also existed between days passed for both *Bacillus* (r_s_ = 0.9270, *p =* 0.0001), and *C. difficile* counts (r_s_ = −0.5138, *p* = 0.0205). The average for each sample from two CFU measurements was used; (**B**) Mice habituating in ‘super clean’ conditions were more susceptible to clindamycin-induced *C. difficile* colonization than ‘conventionally caged’ mice. Mice (n = 5/gp) kept in ‘super clean’ (IVC; sterile food, water and bedding) or ‘conventional cages’ (CC; non-sterile food, water and bedding) for 1 year were challenged with CD630 after dosing with various concentrations of clindamycin. Colonized animals were confirmed by enumerating ethanol-resistant *C. difficile* in cecum 24 h post-challenge. Mice with counts >10^3^ CFU/g were considered colonized. The IC_50_ of IVC (‘super clean’) and CC mice were 0.8 mg/kg and 2.0 mg/kg, respectively; (**C**) *Bacillus* spores were absent in fecal samples of ‘Super clean’ mice. Fecal samples were collected before the challenge study (*panel B*), heated at 65 °C for 45 min., dilutions plated on LB and enumerated after incubation for 2 days at 37 °C. Significance calculated using an unpaired *t*-test. ****, *p* < 0.0001.

Statistical analyses were conducted, and significance determined using the Welch *t*-test for unequal variance unless otherwise specified. All statistical analyses were performed using GraphPad Prism software (v. 9.1.2, Graphpad Software Inc., San Diego, CA, USA).In all experiments involving group comparisons at least six animals were used per group (with the exception of Figure 1A which used five animals per group and Supplementary Figure S8 which used four); for these non-parametric tests it was calculated that a sample size of six per group would be sufficient to detect an effect size of two with 80% power (alpha = 0.05). Spearman rank correlation tests were used to find significant correlations between two continuous variables. When possible, investigators were blinded during group allocation and outcome assessment. Animal groupings were randomized using a random number generator (random.org). Survival functions were estimated using the Kaplan–Meier method and differences analyzed with the log-rank (Mantel–Cox) test using GraphPad Prism software (v.9.1.2).

### 2.20. Data Availability

The data that support the findings of this study are available from the corresponding author.

## 3. Results

### 3.1. C. difficile Colonization Inversely Correlates to the Acquisition of Environmental Bacillus

The acquisition of environmental bacteria has been linked to pathogen susceptibility and colonization [16,17,18,19,27]. As such, we sought to investigate the relationship between allochthonous *Bacillus* spp. and *C. difficile* colonization. Initially, we examined newborn piglet feces for the presence or absence of *Bacillus* spp. and *C. difficile*. This analysis showed that during the first 13 days after birth a declining *C. difficile* population correlated with an increasing *Bacillus* count (*p =* 0.0127) (Figure 1A). Previous work has shown a similar decline of the *C. difficile* population in piglet fecal samples, without postulating a potential mechanism [14,15]. As a microorganism present in the environment, *Bacillus* would be acquired by neonatal pigs accounting for the temporal increase in fecal counts.

In pigs, high hygiene levels have been associated with an increased susceptibility to pathogen colonization and disease [16], raising the intriguing question of whether the same might apply to *C. difficile*. We designed a murine experiment to mimic reduced environmental exposure and then assessed its impact on susceptibility to *C. difficile* colonization. Using mice, we housed animals for 1 year under two standards of hygiene, conventional and ‘super clean’. The former comprised of standard caging (CC, conventional caging), diet, bedding and water used for laboratory animals. ‘Super clean’ involved housing animals in individually ventilated cages (IVCs) that carried HEPA-filtered air together with sterile food, drinking water and bedding; therefore, devoid of microorganisms normally present in food, water and air. In both cases animals were housed individually. To assess susceptibility to *C. difficile* colonization, groups of ‘super clean’ and ‘CC’ mice were administered various concentrations of clindamycin followed by challenge with *C. difficile* (strain CD630, *tcdA^+^ tcdB^+^*) (Figure 1B). Intriguingly, our data showed that ‘super clean’ mice required concentrations of clindamycin sixty percent lower (IC_50_ of 0.8 mg/kg) compared to the CC mice (IC_50_ of 2 mg/kg) to enable *C. difficile* colonization. Analysis of feces from these animals 24 h prior to the administration of clindamycin, to which *Bacillus* is sensitive [37], showed that the ‘super clean’ mice had undetectable levels of *Bacillus*, but in the CC mice counts were between 10^3^ and 10^4^ CFU/g (Figure 1C).

Therefore, animals housed under conditions of reduced environmental exposure (and having depleted fecal *Bacillus* counts (Figure 1C)) were rendered more susceptible to colonization by *C. difficile*. However, ‘super clean’ mice still required prior clindamycin administration and, in its absence, these mice were not colonized by CD630 (Figure 1B). This was not unexpected though since ‘super clean’ conditions are likely to exert a multitude of changes upon the intestinal microbiota, and so any impact caused by the depletion of *Bacillus* on *C. difficile* colonization is unlikely to be absolute, and additional factors may also be contributing to host colonization resistance [12,38]. One possibility is a shift in the microbial composition of the intestinal microbiota in ‘super clean’ mice to genera more susceptible to clindamycin [16].

### 3.2. Environmentally Acquired Bacillus Have Inhibitory Activity towards C. difficile

To further investigate the association between *Bacillus* and *C. difficile* we used standard microbiological methods to isolate *Bacillus* spp. from healthy human, mouse and hamster feces and screened their culture supernatants for direct inhibitory activity to *C. difficile*. Interestingly, the levels of *Bacillus* identified with inhibitory activity against *C. difficile* were roughly equivalent between human (2.4 × 10^3^ CFU/g), mouse (1 × 10^3^ CFU/g) and hamster (3.3 × 10^3^ CFU/g) fecal samples. After clindamycin treatment (30 mg/kg), the number of colonies with inhibitory activity in the feces of mice (6.2 × 10^1^ CFU/g) and hamsters (1.4 × 10^2^ CFU/g) was found to decrease by 10- to 20-fold.

**Figure 2:**
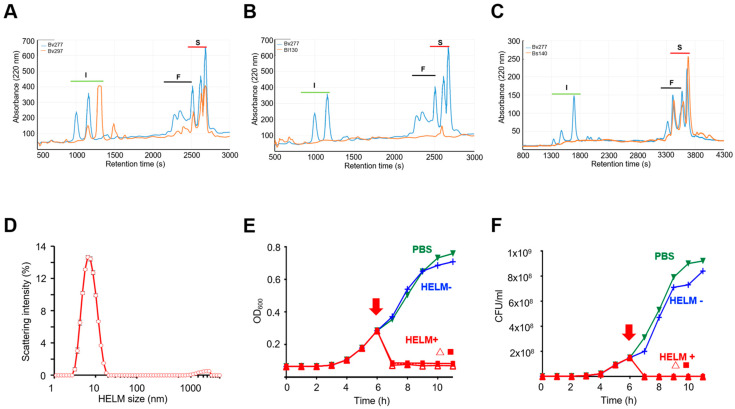
*Bacillus*-produced heterogenous lipopeptide lytic micelles (HELM) particles kill *C. difficile*. (**A**–**C**), SEC fractions from strains of three ‘active’ strains of *Bacillus*: *B. velezensis* strain Bv297 (**A**); *B. licheniformis* strain Bl130 (**B**) and *B. subtilis* strain Bs140 (**C**), were examined by RP-HPLC (orange line). Loading was normalized according to volume of initial culture supernatant. All strains showed inhibitory activity to CD630. Bv297 (**A**) and Bs140 (**C**) were human-derived (Supplementary Table S1) while Bl130 (**B**) was obtained from mouse faeces. The RP-HPLC profile of iturins (I), fengycins (F) and surfactins (S) are indicated and the Bv277 profile (blue line) is shown for comparison. Bl130 showed detectable levels of fengycin and iturin using MALDI-TOF analysis (data not shown); (**D**) HELM particles were analyzed by DLS. Analysis of the Bv277 active SEC fraction revealed the presence of a monodisperse population of micelles with an average diameter of 6.8 ± 0.16 nm (PDI = 0.18). Data points are the average of three measurements with error bars representing the standard error; (**E**,**F**), HELM bacteriolytic activity against CD630. The inhibitory activity to CD630 was determined using measurement of OD600 (**E**) or viable CFU (**F**) before and after addition (arrow) of HELM^+^ (Bv277) culture supernatant (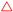), HELM^−^ (*srfAA*-) culture supernatant (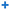), HELM^+^ (Bv277) SEC-fractionated ‘active’ fraction (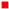) and untreated (PBS) (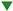).

Previous taxonomic profiling of ‘active’ murine, human and hamster isolates revealed them to be three species of *Bacillus* (*B. subtilis* (Bs), *B. velezensis* (Bv) and *B. licheniformis* (Bl)) [23], which was confirmed here (Supplementary Table S1). Bioanalysis of the active component within the culture filtrates of inhibitory *Bacillus* spp. revealed it to be a high MW micellar complex comprised of various lipopeptides that together were capable of lytic activity against *C. difficile* [23]. This complex is henceforth referred to as a HELM (heterogenous lipopeptide lytic micelle). Previous work has shown this HELM to be comprised of the cyclic lipopeptides, iturin, fengycin, surfactin (three isoforms) [39] and the dipeptide antibiotic chlorotetaine [23,40]. This was confirmed here using RP-HPLC and MALDI-TOF analysis of Bv277 material, which was selected as an exemplar due to the high level of inhibitory activity it demonstrated against *C. difficile* (Supplementary Figure S2A, Supplementary Tables S1 and S2). All individual compounds inhibited growth of CD630 (Supplementary Figure S2B). Combining them together further enhanced their inhibitory effect, which exceeded the expected additive effect of the combined fractions by ~2-fold, indicating a synergistic action (Supplementary Figure S2B). Accordingly, for inhibitory *Bacillus* strains we found that the highest activity was associated with the presence of all four compounds (Supplementary Table S1, Supplementary Figure S2B). Representative, inhibitory strains from each of the three species of *Bacillus* were found to produce detectable levels of HELMs, as confirmed by RP-HPLC and MALDI-TOF analysis (Figure 2A–C). Supernatants from two representative strains with no activity against *C. difficile* from *B. velezensis* (Bv378) and *B. subtilis* (PY79) showed no detectable lipopeptides (Supplementary Figure S3). Dynamic light scattering (DLS) analysis further confirmed that *Bacillus* HELMs were forming mixed micelle particles, showing monodisperse and stable micelles of ~6.8 nm (Figure 2D).

To demonstrate the importance of HELMs for *Bacillus* inhibitory activity against *C. difficile*, we constructed a mutant carrying an insertion in the *srfAA* gene that is involved in the biosynthesis of surfactin (Supplementary Figure S4, Supplementary Methods) [41]. The cell-free supernatant of the Bv277 *srfAA* mutant (HELM^−^) produced no surfactins, biosurfactant activity and in vitro activity (Figure 2E,F, Supplementary Figure S4A). Interestingly, although present, levels of iturin and fengycin appeared to be reduced in the *srfAA* mutant suggesting possible disruption to the HELM complex due to the absence of the principal component, surfactin (Supplementary Figure S4C).

Addition of the Bv277 HELM^+^ supernatant to growing cultures of CD630 showed lysis with a rapid reduction in OD_600_ and viable CFU confirming a bacteriolytic action, whilst supernatant from the Bv277 *srfAA* mutant (HELM^−^) showed no such activity (Figure 2E,F, Video S1). This is consistent with the mechanism of action of lipopeptides; via hydrophobic interactions, penetration and pore formation of biological membranes resulting in permeability changes and cell lysis [42]. Taken together, our data demonstrate that *Bacillus*-produced lipopeptides, coalesced as HELM particles, with inhibitory activity against *C. difficile*.

### 3.3. HELMs in the SI Can Inhibit C. difficile

**Figure 3:**
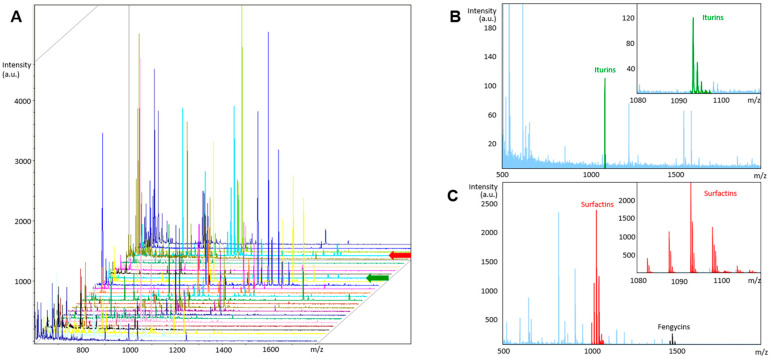
Detection of HELMs in the SI. Panel A, MALDI-TOF analysis of 30 collected individual fractions following RP-HPLC analysis of pooled SI contents from naive mice (n = 3) (Supplementary Figure S5). Lipopeptides were identified in four fractions and representative mass spectra (colored arrows) are shown (**B**,**C**); (**B**) Representative MALDI-TOF spectra of an individual fraction (green arrow in **A**) containing iturins (highlighted in green). Displayed is a zoomed in mass spectrum of the detected C16IturinA/C16Mycosubtilin/C15BacillomycinF; (**C**) Representative MALDI-TOF spectra of an individual fraction (red arrow in **A**) containing surfactins (red) and fengycins (black). Displayed is a zoomed in mass spectrum of the detected surfactins (C12 to C15). Light blue color shows unidentified masses.

We next investigated whether HELMs produced by environmentally acquired *Bacillus* spp. could be detected within the GI-tract. *Bacillus* spores have been shown to germinate and proliferate in the SI [19] after which they secrete extracellular compounds such as toxins, as is the case with *B. cereus* [43]. We therefore employed RP-HPLC and MALDI-TOF analysis to detect the presence of lipopeptides in the SI of naïve mice, or mice with depleted intestinal *Bacillus* CFU, post clindamycin administration. Methanol extraction of SI samples followed by analysis of the intestinal contents of naïve mice confirmed the presence of HELMs (Figure 3A–C), which diminished to undetectable levels in mice administered with clindamycin. No lipopeptides could be detected in the feces of naïve or clindamycin-treated mice (data not shown).

The SI is also the site of attachment for many pathogens, including *C. difficile* that colonizes its host by attaching to the epithelial mucosa [44]. Interestingly, studies have shown that germination of *C. difficile* spores was equivalent within the SI of mice with or without prior antibiotic treatment [45], which we verified both ex vivo and in vivo (Supplementary Figure S6). It follows then, that in the GI-tract of healthy individuals, various colonization resistance factors must target germinated *C. difficile* spores and thereby impair disease progression.

**Figure 4:**
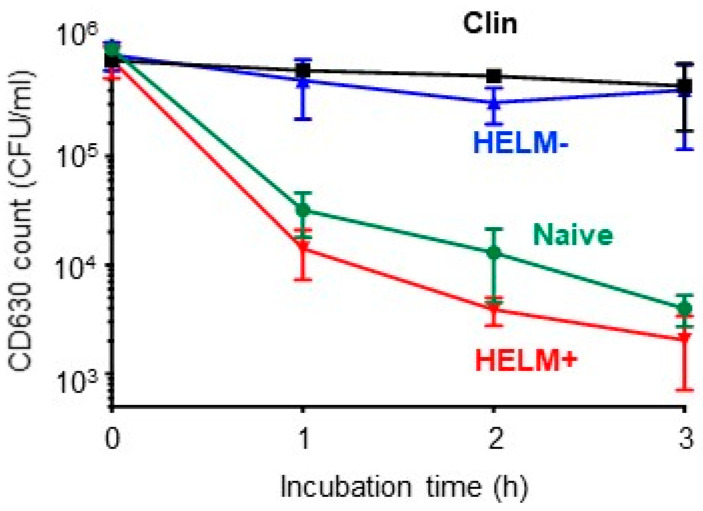
HELMs in the SI can inhibit *C. difficile*. Ex vivo analysis revealed CD630 inhibitory activity in the SI-extract of naïve and HELM^+^ (Bv277)-dosed mice. CD630 inhibitory activity was determined using methanol extracts taken from the SI contents of mice (n = 3) with or without clindamycin treatment (Clin and Naive). The contents were taken from the SI of mice treated with clindamycin followed by oral dosing with spores (2 × 10^9^ CFU) of HELM^+^ (Bv277) or HELM^−^ (*srfAA*-). The experiment was performed twice independently. Error bars represent standard deviation.

For *Bacillus*-HELMs to contribute to colonization resistance, a reasonable assumption is that this would occur in the SI, the site of *Bacillus* proliferation, HELM production and C. difficile germination. To test this, clindamycin-treated mice (with depleted intestinal *Bacillus* CFU) were dosed with HELM^+^ (Bv277) or HELM^−^ (*srfAA*-) *Bacillus* spores after which the SI contents were collected, and methanol extracted. Extracts were reconstituted in PBS and incubated (4 °C, 18 h) to allow micellar assembly of amphiphilic components after which they were incubated with CD630 spores in BHIS medium containing 1% sodium taurocholate to induce *C. difficile* germination. Extracts derived from HELM^+^-dosed mice inhibited germinated *C. difficile* cells, while those from HELM^−^-dosed animals did not (Figure 4). SI extracts from naïve mice also showed similar inhibition of CD630 to that of HELM^+^-dosed mice, while extracts from clindamycin-dosed mice showed no inhibition. Clearly the compounds present within the SI of naïve and HELM^+^-dosed mice exhibit an inhibitory effect on *C. difficile*. These compounds must therefore be absent in the antibiotic- and HELM^−^-dosed mice. This suggests a mechanism whereby the production of HELMs by inhibitory *Bacillus* species can mediate killing of germinated *C. difficile* in the SI. This is further supported by the lack of activity observed in antibiotic-treated mice, suggesting that the production of inhibitory compounds is reduced following clindamycin depletion of *Bacillus*. Our results differ somewhat from that of a previous study which failed to observe a killing effect of untreated SI-contents [45] and we suspect that the more rigorous (methanol) extraction procedure used here may account for this difference. Methanol extraction is likely to solubilize a large number of hydrophobic molecules, potentially those with activity against *C. difficile* [42], including antimicrobials and secondary bile acids.

### 3.4. Synergistic Activity of HELMs

**Figure 5:**
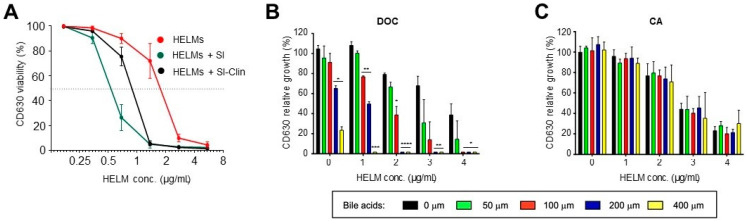
HELMs synergize with secondary bile acids. (**A**) HELMs synergize with SI extracts taken from mice. A sterile, cell-free Bv277 supernatant was incubated (3 h, RT) 1:1 with: PBS (HELMs) or methanol extracts in saline (1/3, *w*/*v*) taken from a naïve mouse SI (HELMs + SI) or SI taken from a mouse treated 24 h previously with 30 mg/kg clindamycin (HELMs + SI-Clin). The mixtures were serially diluted and inhibitory activity to CD630 was measured using the microdilution assay. At the dilution factors used in this experiment no activity was observed with the SI or SI-Clin extract only. The experiment was performed twice independently. Error bars represent standard deviation; (**B**,**C**), HELMs synergize with deoxycholate. Various dilutions of a sterile, cell-free Bv277 supernatant (containing 1 to 4 μg/mL HELMs) were mixed with different concentrations (50–400 μM) of secondary bile acid deoxycholate (DOC) (**B**) or primary bile acid cholate (CA) (**C**) and added to mid-logarithmic cultures of CD630 and incubated for 5 h at 37 °C. OD readings (600 nm) were taken, and relative growth determined as the percentage increase in OD600 from the time of addition of samples to CD630 culture. The experiment was performed twice independently. Error bars represent standard deviation. Significance calculated using an unpaired *t*-test. *, *p* < 0.05, **, *p* < 0.01, ***, *p* < 0.001, ****, *p* < 0.0001.

Resistance to enteric pathogen colonization is most likely an amalgamation of various mechanisms involving bile acids, bacteriophages, nutrient competition, SCFAs and bacteriocins [46]. This functional redundancy may help to explain why no single factor can provide complete colonization resistance, and why the combination of several contributory factors is necessary for disease prevention. Indeed, a recent study provides evidence that intestinal proliferation of *C. difficile* is regulated by synergy between secondary bile acids and microbiota-produced antimicrobials [46]. Therefore, we asked whether *Bacillus* HELMs could form part of this dynamic by enabling interaction with other components within the GI-tract to augment host colonization resistance against *C. difficile*. To determine whether HELMs could enhance inhibitory activity to *C. difficile* when combined with the contents of the GI-tract, we first incubated HELMs with SI-methanol extracts taken from mice with or without clindamycin treatment, followed by analysis of their inhibitory activity using a microplate assay (Figure 5A). When SI-extracts were added to HELMs, the HELM concentration required to decrease the viability of a *C. difficile* culture to less than 50% was reduced. This could result either from the presence of bile acids in the intestinal tract, mouse-derived HELMs, antimicrobials or hitherto unknown molecules. This finding is not necessarily surprising as there is extensive evidence for the ability of lipopeptides to synergize with other antimicrobial agents [47,48]. For SI extracts from clindamycin-treated mice, activity was increased but less so than with the naive sample and might be explained if resident microbiota had been killed, resulting in lower levels of antimicrobials, HELMs or bile salts.

Secondary bile acids have also been shown to synergize with antimicrobials in the GI-tract [49,50,51] and they share many physiochemical properties with HELM lipopeptides [48,52]. We speculated that HELM inhibitory activity to *C. difficile* might be augmented by synergistic interaction with bile acids. Although secondary bile acids are mostly found in the large intestine, significant quantities have also been measured in the SI [53,54]. We observed a strong concentration-dependent enhancement of inhibition (synergy) against *C. difficile* by HELMs when combined with deoxycholate (DOC) (Figure 5B). By contrast, the same experiment using the primary bile acid cholic acid (CA) showed no effect (Figure 5C). Taken together, these data demonstrate that HELMs and DOC synergize with one another, thereby increasing antimicrobial activity.

Synergy between bile acids and HELMs likely results from the complementarity of physio-chemical properties and the hydrophilic–hydrophobic balance arising from interactions between different amphiphiles (Supplementary Figure S5). This ability to disrupt lipid membranes correlates with increases in the hydrophobicity, interfacial and micellar properties of amphiphiles, and most probably accounts for the increased antibacterial activity of bile acid–HELM combinations.

### 3.5. Exclusion of C. difficile Colonization by HELM-Producing Bacillus

**Figure 6:**
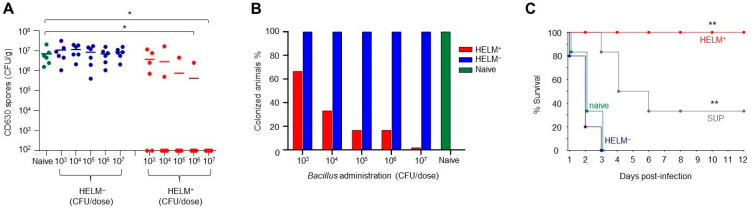
Exclusion of *C. difficile* colonization by HELM-producing *Bacillus*. (**A,B**) Bv-HELM^+^ spores inhibit *C. difficile* colonization in a dose-dependent manner using a microbiota depletion model. Mice (n = 6/gp) were treated with an antibiotic cocktail for 7 days, followed by a ‘washout’ period of 2 days before being challenged with 10^3^ CD630 spores. Mice were dosed three times daily with spores of either 10^7^, 10^6^, 10^5^, 10^4^ or 10^3^ CFU/dose of HELM^+^ (Bv277), HELM^−^ (*srfAA*-) or PBS (naïve) during the two washout days and on the day of challenge. At 24 h post-challenge, ceca were removed for analysis of levels of ethanol-resistant spore counts (**A**) and toxin A (Supplementary Figure S7A). The percentage of colonized animals was calculated as the proportion of mice within each group with *C. difficile* spores (>10^3^ CFU/g) and toxin present within the ceca (**B**). Significance was calculated using an unpaired *t*-test. *, *p* < 0.05; (**C**) Bv-HELM^+^ spores inhibit *C. difficile* colonization in a hamster clindamycin model. Golden Syrian hamsters were administered clindamycin and 13 h later dosed 3-times/day (6 h intervals) orally (i.g.) with ~2 × 10^9^ CFU/dose of HELM^+^ (Bv277), HELM^−^ (Bv378) or the cell-free supernatant of HELM^+^ (2 mL/dose) until study end. At 72 h post-clindamycin treatment animals were challenged with 10^2^ spores of CD630. Animal survival is shown and toxin and CFU analysis is shown in Supplementary Figure S6A,B. Significance was tested with the log-rank (Mantel–Cox) test. **, *p* < 0.01.

To discern the causal effect that HELM-producing *Bacillus* exerts upon *C. difficile* colonization in lieu of other contributing factors, we used a microbiota depletion model where animals are subject to a prolonged multi-antibiotic treatment [55,56]. This approach offers several advantages over a germ-free model, including, but not limited to, the detrimental effect long-term sterility has upon mouse development and immune function [56]. To deplete the pre-existing intestinal microbiota, mice were first treated with an antibiotic cocktail for one week followed by a washout period of two days. During the washout period mice were orally dosed with a range of concentrations of HELM^+^ or HELM^−^ spores (10^3^ to 10^7^ CFU/dose). The dosages used in this experiment encompass the physiological levels of *Bacillus* found in the intestine of both mice and humans (~10^4^ to 10^5^ CFU/g) [57]. Higher doses were also used (~10^6^ to 10^7^ CFU/g), as, unlike in a healthy host, HELM-producing *Bacillus* would be the sole colonization resistance factor present in this model. On the third day post-antibiotic cessation, mice were challenged with 10^3^ CD630 spores and dosing with HELM^+^ or HELM^−^ spores continued (Figure 6A,B, Supplementary Figure S7A). Analysis of the cecum for correlates of infection showed that the administration of HELM-producing spores prevented *C. difficile* colonization in a dose-dependent manner. Dosing with 10^7^ CFU HELM^+^ spores prevented colonization in all mice, with no *C. difficile* toxin or CFU observed. Mice dosed with 10^6^, 10^5^ and 10^4^ CFU of HELM^+^ spores demonstrated high levels of colonization resistance, while animals dosed with 10^3^ CFU displayed lower levels. By contrast, administration of HELM^−^ spores had no effect on colonization, regardless of dosage, demonstrating the necessity of HELMs in colonization resistance. To further assess the role of HELM-producing *Bacillus* in *C. difficile* colonization resistance, we used a clindamycin challenge model in Golden Syrian hamsters (Figure 6C, Supplementary Figure S7B,C). This hamster model was chosen to mimic the infection process in humans and assess the capability of *Bacillus* HELMs to prevent colonization. When hamsters were administered HELM^+^ spores, they showed complete protection against CDI with all animals exhibiting no symptoms of disease 12 days post-challenge together with the absence of both toxins and *C. difficile* in the ceca (Figure 6C, Supplementary Figure S7B,C). Hamsters dosed with a HELM^−^ strain (Bv378) having no in vitro activity were not protected, with kinetics similar to naive animals. Hamsters dosed with the cell-free HELM^+^ supernatant showed a delayed onset of CDI symptoms with 2/6 animals surviving.

These in vivo studies demonstrate that exclusion of *C. difficile* colonization is associated with the consumption of HELM-producing *Bacillus*. The administration of these *Bacilli* at the approximate quantity as that found in the GI-tract of mice and humans is sufficient to preclude CDI by preventing host colonization. Further in vivo studies in mice and hamsters have been conducted which support this conclusion, including the use of other clinically relevant *C. difficile* ribotypes (027, 078) (Supplementary Figure S8). Taken together, this suggests that environmentally acquired HELM producing *Bacillus* play an integral role in resistance to *C. difficile* colonization.

## 4. Discussion

Our work provides important insights into the impact that environmentally acquired *Bacillus* have upon *C. difficile* colonization. Specifically, exposure to these allochthonous bacteria contributes to colonization resistance, and, conversely, reduced exposure may therefore increase susceptibility to disease. Remarkably, as shown here, the relative abundance of ‘inhibitory’ *Bacillus* spp. in the GI-tracts of humans and animals is essentially equivalent indicating steady-state population dynamics. We show that *Bacillus* spp. are able to efficiently kill *C. difficile* by producing cyclic lipopeptides that under favorable conditions, such as those found in the GI-tract, form novel micelle particles which we refer to as HELMs. *Clostridia* and *Bacillus* spp. have evolved to compete in a shared soil environment [20,58]. It is therefore not surprising that *Bacillus* has direct inhibitory activity towards *Clostridia* spp., nor that this competition could be exported to the GI-tract, another shared environment. Surprisingly, we have also found that HELM particles can synergize with other compounds present in the GI-tract increasing inhibitory activity further still. Current therapies for *C. difficile* are not ideal, and the work here provides a potential link between exposure to environmental bacteria and *C. difficile* susceptibility and could act as a foundation for future work for prophylactic treatment of CDI.

*Bacillus*-produced cyclic lipopeptides are associated with a plethora of important functions including antagonism against other microorganisms [59], biofilm development [60], motility [61] and cell-surface attachment [62]. Non-ribosomal lipopeptide biosynthesis is heavily influenced by pH, temperature, nitrogen and carbohydrate availability [63], and antibacterial potency is dependent on the presence of other amphiphilic compounds with which they can co-associate to form mixed micelles [42]. An important finding made here is the demonstration that the SI provides a suitable environment for HELMs to inhibit *C. difficile* outgrowth and, considering the amphiphilic and synergistic nature of lipopeptides, it is probable that this occurs via the formation of mixed micelles. We have also found that the composition of HELM micelles can differ with the most potent formulations incorporating antibiotics (exampled here is chlorotetaine) [23]. We would predict this to be a more general phenomenon linked to micelle formulation and we would anticipate that other antimicrobial compounds might also be incorporated into HELMs.

Bacteriocins and antimicrobials readily synergize with one another [64], and there is evidence for mixed micellization and synergy between bile acids and antimicrobial agents against pathogens [49,50], including *C. difficile* [51]. Microbiota-derived antimicrobial peptides have been shown to be key factors in regulating intestinal bacterial populations [65]. Colonization resistance is known to be a complex phenomenon, and accumulating evidence suggests that a crucial aspect is synergism between bacterially produced agents [51,65,66]. Considering that *Bacillus* in vitro lipopeptide production and subsequent solubility is dependent on nutrient and mineral availability [63], it is not inconceivable that the abundance and composition of intestinal HELMs is also affected by diet. Furthermore, the abundance of HELMs in the GI-tract is likely to impact upon the solubility, stability and antimicrobial activity of other intestinal components, such as secondary bile acids [67]. If found to be correct, this may play a part in explaining why different individuals exhibit differing levels of colonization resistance.

Antibiotic-mediated dysbiosis of the gut microbiota has been linked to a reduction in *Clostridium* spp. and other 7 α-dehydroxylating bacteria, resulting in decreased levels of secondary bile acids and a subsequent decline in resistance to CDI [11]. A notable example is *C. scindens*, that has been shown to transform primary bile acids (e.g., CA) to *C. difficile*-inhibitory secondary bile acids (e.g., DOC), thereby conferring resistance to CDI [12]. The important, but limited, role that *C. scindens* plays in colonization resistance has also been observed using a simplified murine 12-species oligo-mouse microbiota model [68]. It is probable that colonization resistance is founded upon an interplay between different factors that build resistance and prevent outgrowth of pathogens. One example would be the regulation of *C. difficile* growth by synergy between microbiota-produced antibiotics and secondary bile acids [51]. We suggest then, that although colonization resistance is mediated by several factors, important among them is the combined synergistic action of HELMs produced by *Bacillus* species and other soluble antimicrobial compounds [69], including secondary bile acids.

Studies over the last few decades suggest that changing human lifestyles has affected our exposure to allochthonous bacteria, a grouping that will include *Bacillus* [70]. Rural populations have a higher exposure to environmental microbes and a more diverse gut microbiota [71,72]. On the other hand, increased urbanization has reduced microbial diversity [73]. A consequence of this has been a reduction in exposure to soil microorganisms and a less diverse gut microbiota [74]. The process of urbanization therefore results in higher inter-individual variation of gut microbial species, a finding which would likely extend to *Bacillus* species. This might be one factor that could account for the pronounced increase in CDI witnessed over the last 30 years [2], the appearance of CDI in infants [3,75] and also community-associated CDI, where up to 50% of CDI cases (albeit less severe) are reported to have arisen from outside of the hospital [76]. Three recent studies have illustrated links between a ‘Western diet’ and susceptibility to, or severity of, CDI. A fiber-deficient diet has been linked to increased susceptibility to CDI [38], while increased consumption of sugars has been driving speciation of more virulent variants of *C. difficile* [77] and has impacted upon the emergence of hypervirulent strains [78]. It is becoming apparent that our exposure to environmental bacteria may also be impacting our susceptibility to pathogens. Indeed, a case in point is a recent study showing the importance of *Bacillus* fengycins in controlling growth of *S. aureus* in the GI-tract [27]. Considering that both *Bacillus* and *Clostridia* are soil-borne, the findings from this study suggest that the evolutionary competition begun in the soil by the progenitors of these two genera may have resumed within the human microbiome [79,80]. An increased focus on these environmentally acquired bacteria may provide future measures to control CDI.

## Data Availability

The data presented in this study are available from the corresponding author upon reasonable request.

## Supplementary Materials

The following supporting information can be downloaded at: https://www.mdpi.com/article/10.3390/biomedicines10050930/s1, Figure S1: Study schematic; Figure S2: RP-HPLC analysis of HELMs from Bv277; Figure S3: RP-HPLC analysis of HELMs from negative Bacillus isolates; Figure S4: Construction of a Bv277 srfAA mutant (HELM−); Figure S5: RP-HPLC analysis of HELMs present in the small intestine; Figure S6: *C. difficile* germinates in the presence or absence of clindamycin within the GI-tract; Figure S7: Studies in mice and Golden Syrian hamsters; Figure S8: Effect of HELM^+^ on colonization of ribotypes 027 and 078; Table S1: Human Bacillus strains with inhibitory activity to *C. difficile*; Table S2: MALDI-TOF analysis of Bv277 HELM RP-HPLC active fractions; Video S1: Bv277 lysis of *C. difficile*; Supplementary methods [81,82,83].
