# Supplementary material for: Environmentally Acquired Bacillus and Their Role in C. difficile Colonization Resistance
Source: Biomedicines. 2022 Apr 19;10(5):930. doi: 10.3390/biomedicines10050930 (PMC9138776; doi:10.3390/biomedicines10050930)
Supplement: Supplementary file 1 [file biomedicines-10-00930-s001.zip › Supplementary Figures.pptx]

## Slide 1
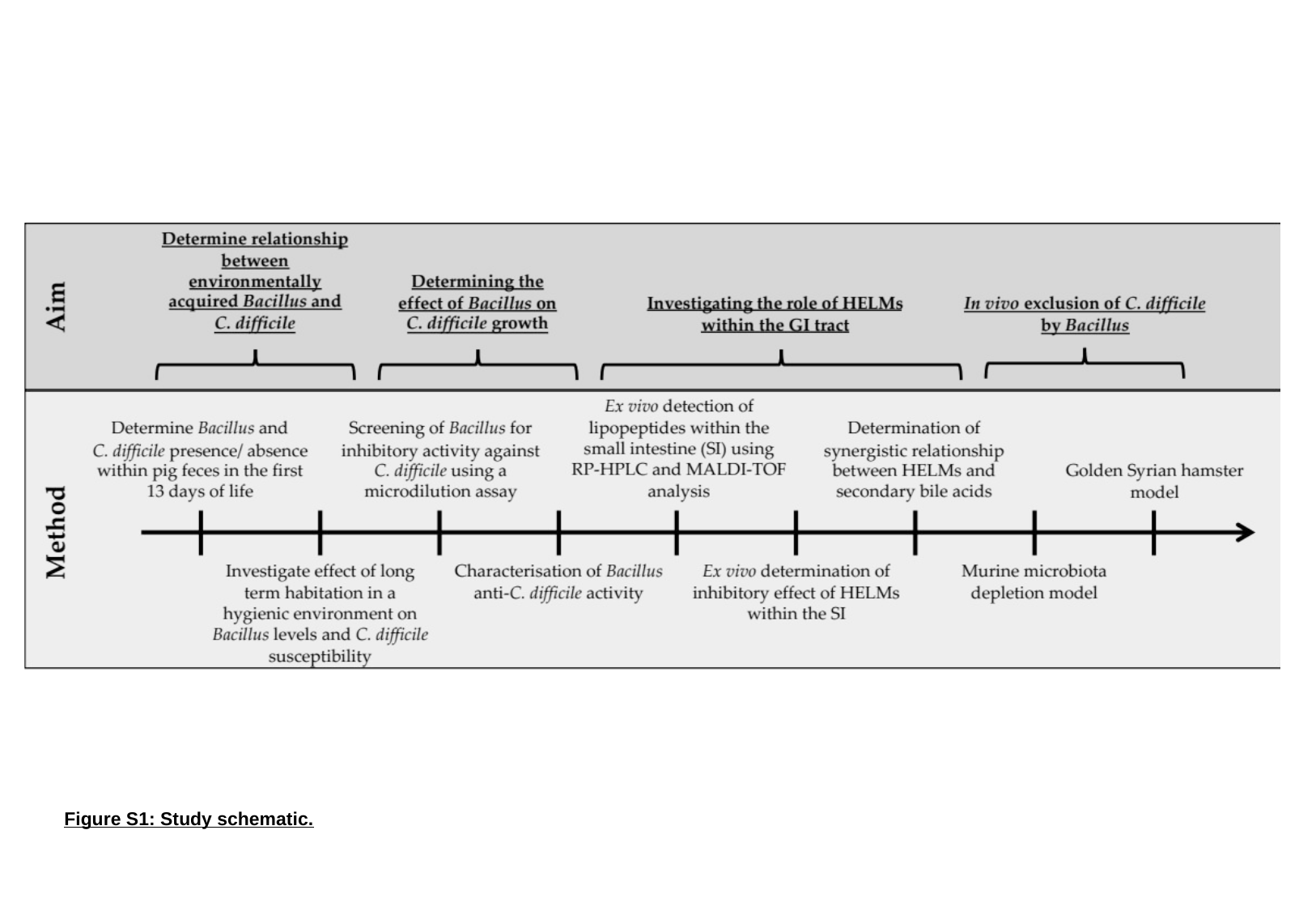

Figure S1: Study schematic.

## Slide 2
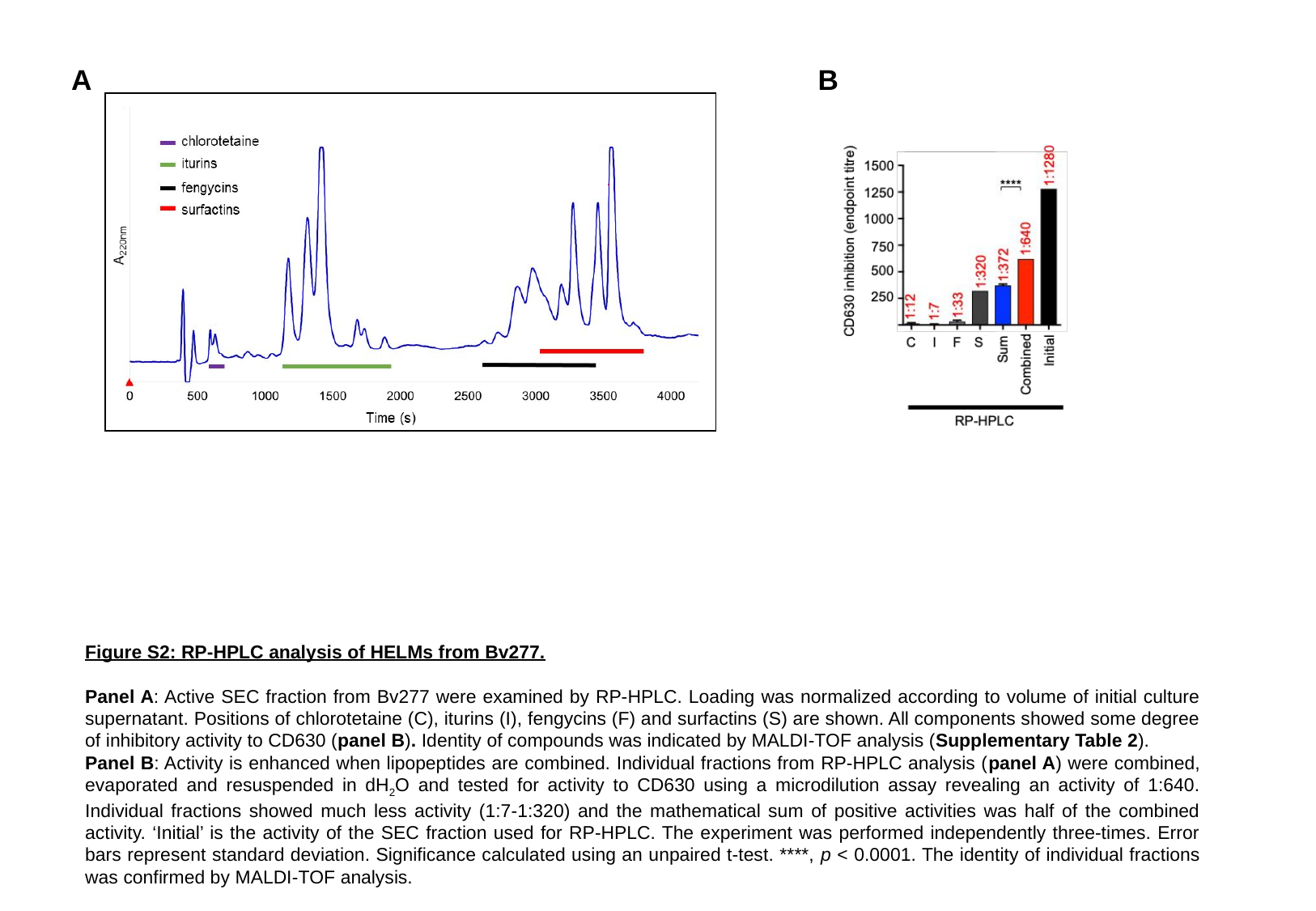

A
B
Figure S2: RP-HPLC analysis of HELMs from Bv277.
Panel A: Active SEC fraction from Bv277 were examined by RP-HPLC. Loading was normalized according to volume of initial culture supernatant. Positions of chlorotetaine (C), iturins (I), fengycins (F) and surfactins (S) are shown. All components showed some degree of inhibitory activity to CD630 (panel B). Identity of compounds was indicated by MALDI-TOF analysis (Supplementary Table 2).
Panel B: Activity is enhanced when lipopeptides are combined. Individual fractions from RP-HPLC analysis (panel A) were combined, evaporated and resuspended in dH2O and tested for activity to CD630 using a microdilution assay revealing an activity of 1:640. Individual fractions showed much less activity (1:7-1:320) and the mathematical sum of positive activities was half of the combined activity. ‘Initial’ is the activity of the SEC fraction used for RP-HPLC. The experiment was performed independently three-times. Error bars represent standard deviation. Significance calculated using an unpaired t-test. ****, p < 0.0001. The identity of individual fractions was confirmed by MALDI-TOF analysis.

## Slide 3
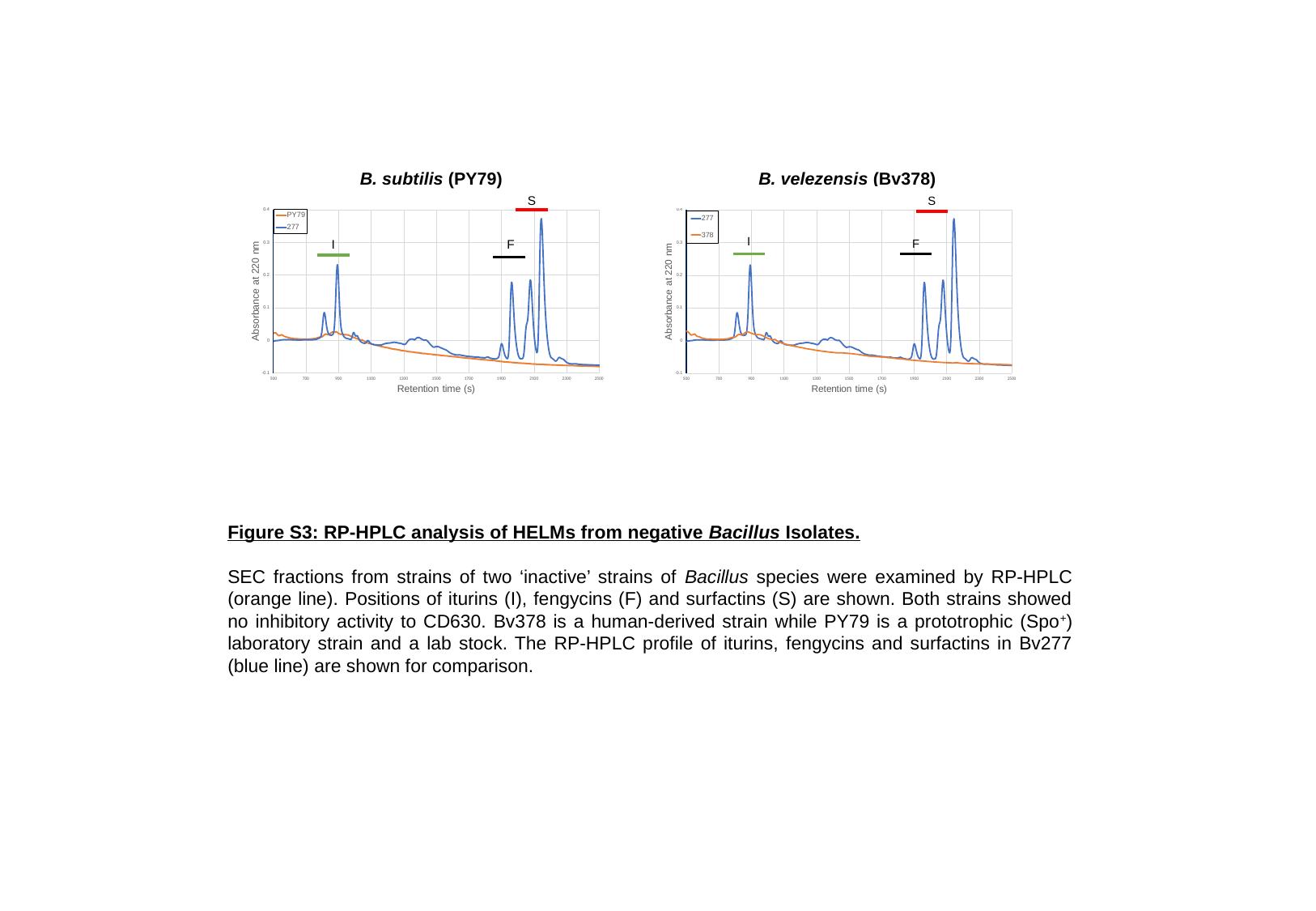

B. subtilis (PY79)
B. velezensis (Bv378)
Figure S3: RP-HPLC analysis of HELMs from negative Bacillus Isolates.
SEC fractions from strains of two ‘inactive’ strains of Bacillus species were examined by RP-HPLC (orange line). Positions of iturins (I), fengycins (F) and surfactins (S) are shown. Both strains showed no inhibitory activity to CD630. Bv378 is a human-derived strain while PY79 is a prototrophic (Spo+) laboratory strain and a lab stock. The RP-HPLC profile of iturins, fengycins and surfactins in Bv277 (blue line) are shown for comparison.

## Slide 4
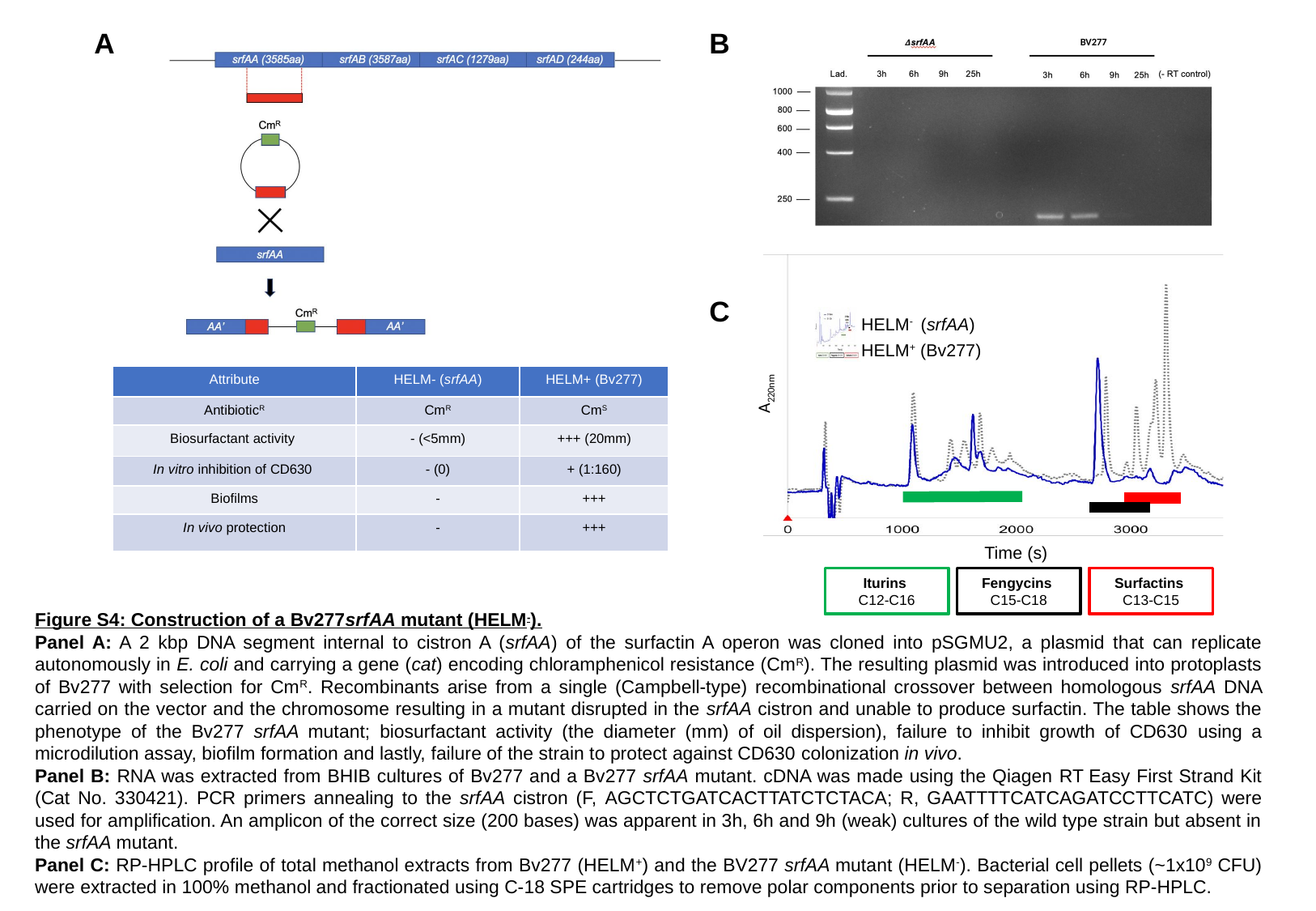

A
B
BV277
C
HELM- (srfAA)
HELM+ (Bv277)
A220nm
| Attribute | HELM- (srfAA) | HELM+ (Bv277) |
| --- | --- | --- |
| AntibioticR | CmR | CmS |
| Biosurfactant activity | - (<5mm) | +++ (20mm) |
| In vitro inhibition of CD630 | - (0) | + (1:160) |
| Biofilms | - | +++ |
| In vivo protection | - | +++ |
Time (s)
Iturins
C12-C16
Fengycins
C15-C18
Surfactins
C13-C15
Figure S4: Construction of a Bv277srfAA mutant (HELM-).
Panel A: A 2 kbp DNA segment internal to cistron A (srfAA) of the surfactin A operon was cloned into pSGMU2, a plasmid that can replicate autonomously in E. coli and carrying a gene (cat) encoding chloramphenicol resistance (CmR). The resulting plasmid was introduced into protoplasts of Bv277 with selection for CmR. Recombinants arise from a single (Campbell-type) recombinational crossover between homologous srfAA DNA carried on the vector and the chromosome resulting in a mutant disrupted in the srfAA cistron and unable to produce surfactin. The table shows the phenotype of the Bv277 srfAA mutant; biosurfactant activity (the diameter (mm) of oil dispersion), failure to inhibit growth of CD630 using a microdilution assay, biofilm formation and lastly, failure of the strain to protect against CD630 colonization in vivo.
Panel B: RNA was extracted from BHIB cultures of Bv277 and a Bv277 srfAA mutant. cDNA was made using the Qiagen RT Easy First Strand Kit (Cat No. 330421). PCR primers annealing to the srfAA cistron (F, AGCTCTGATCACTTATCTCTACA; R, GAATTTTCATCAGATCCTTCATC) were used for amplification. An amplicon of the correct size (200 bases) was apparent in 3h, 6h and 9h (weak) cultures of the wild type strain but absent in the srfAA mutant.
Panel C: RP-HPLC profile of total methanol extracts from Bv277 (HELM+) and the BV277 srfAA mutant (HELM-). Bacterial cell pellets (~1x10^9^ CFU) were extracted in 100% methanol and fractionated using C-18 SPE cartridges to remove polar components prior to separation using RP-HPLC.

## Slide 5
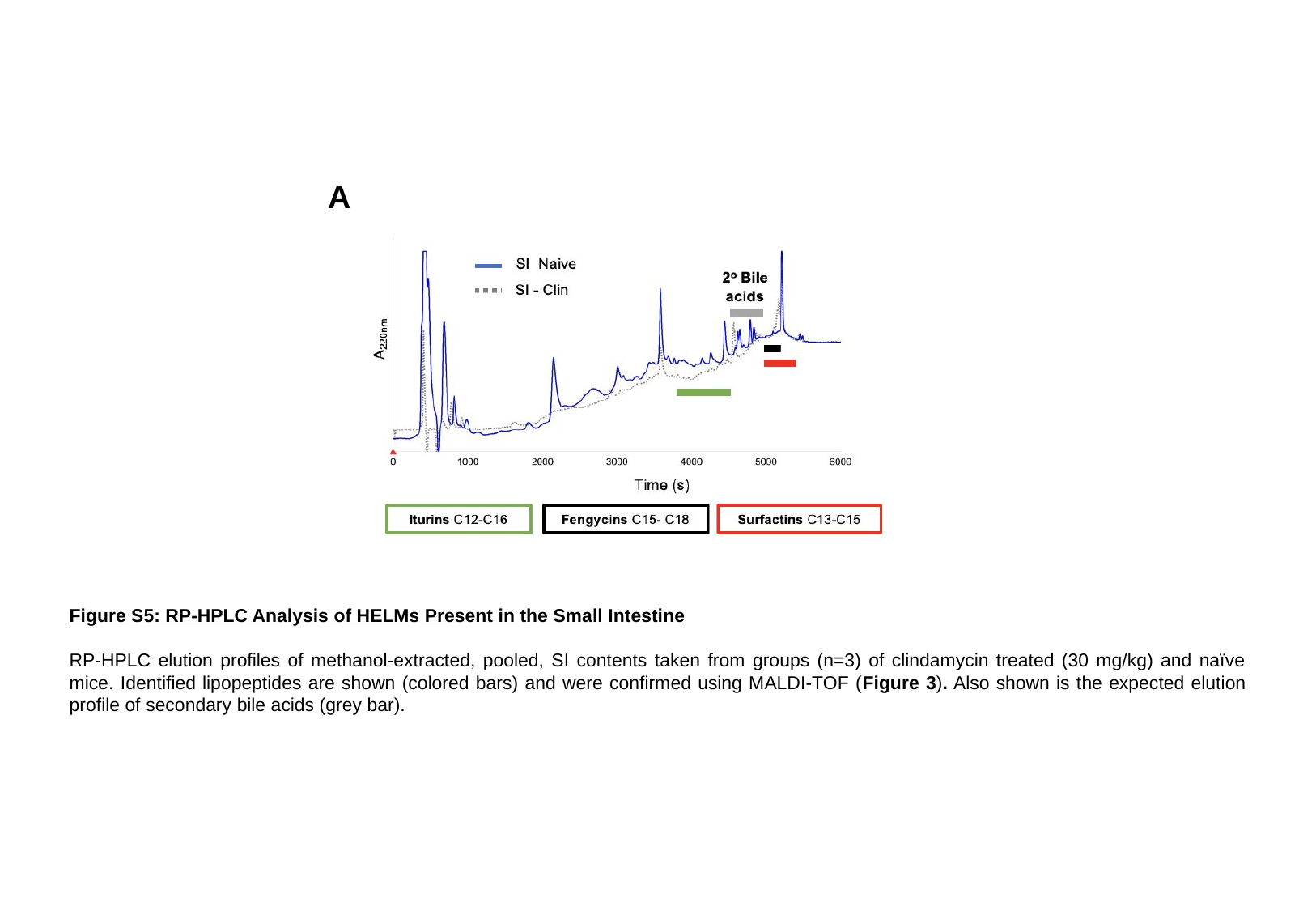

A
Figure S5: RP-HPLC Analysis of HELMs Present in the Small Intestine
RP-HPLC elution profiles of methanol-extracted, pooled, SI contents taken from groups (n=3) of clindamycin treated (30 mg/kg) and naïve mice. Identified lipopeptides are shown (colored bars) and were confirmed using MALDI-TOF (Figure 3). Also shown is the expected elution profile of secondary bile acids (grey bar).

## Slide 6
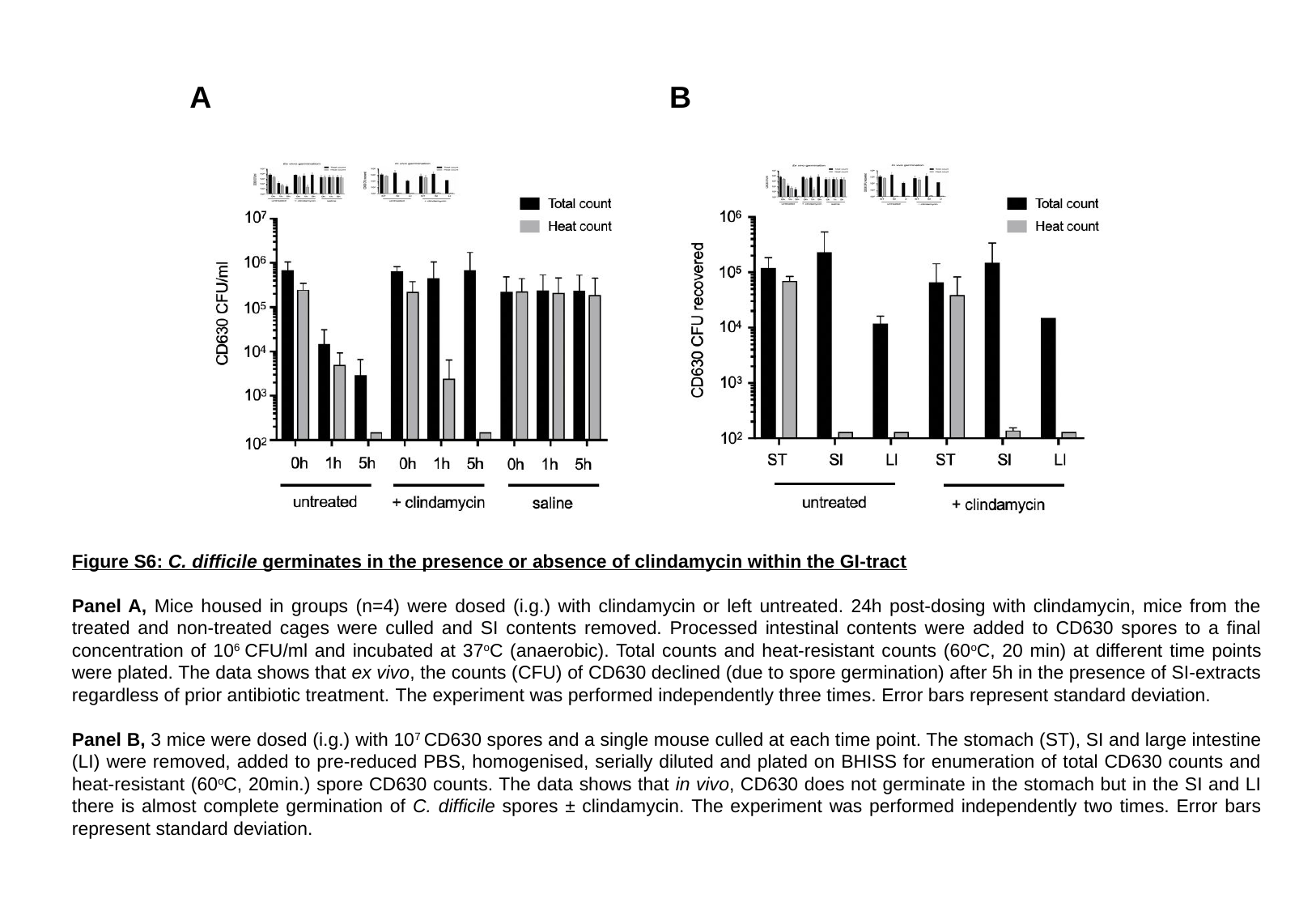

A
B
Figure S6: C. difficile germinates in the presence or absence of clindamycin within the GI-tract
Panel A, Mice housed in groups (n=4) were dosed (i.g.) with clindamycin or left untreated. 24h post-dosing with clindamycin, mice from the treated and non-treated cages were culled and SI contents removed. Processed intestinal contents were added to CD630 spores to a final concentration of 106 CFU/ml and incubated at 37oC (anaerobic). Total counts and heat-resistant counts (60oC, 20 min) at different time points were plated. The data shows that ex vivo, the counts (CFU) of CD630 declined (due to spore germination) after 5h in the presence of SI-extracts regardless of prior antibiotic treatment. The experiment was performed independently three times. Error bars represent standard deviation.
Panel B, 3 mice were dosed (i.g.) with 107 CD630 spores and a single mouse culled at each time point. The stomach (ST), SI and large intestine (LI) were removed, added to pre-reduced PBS, homogenised, serially diluted and plated on BHISS for enumeration of total CD630 counts and heat-resistant (60oC, 20min.) spore CD630 counts. The data shows that in vivo, CD630 does not germinate in the stomach but in the SI and LI there is almost complete germination of C. difficile spores ± clindamycin. The experiment was performed independently two times. Error bars represent standard deviation.

## Slide 7
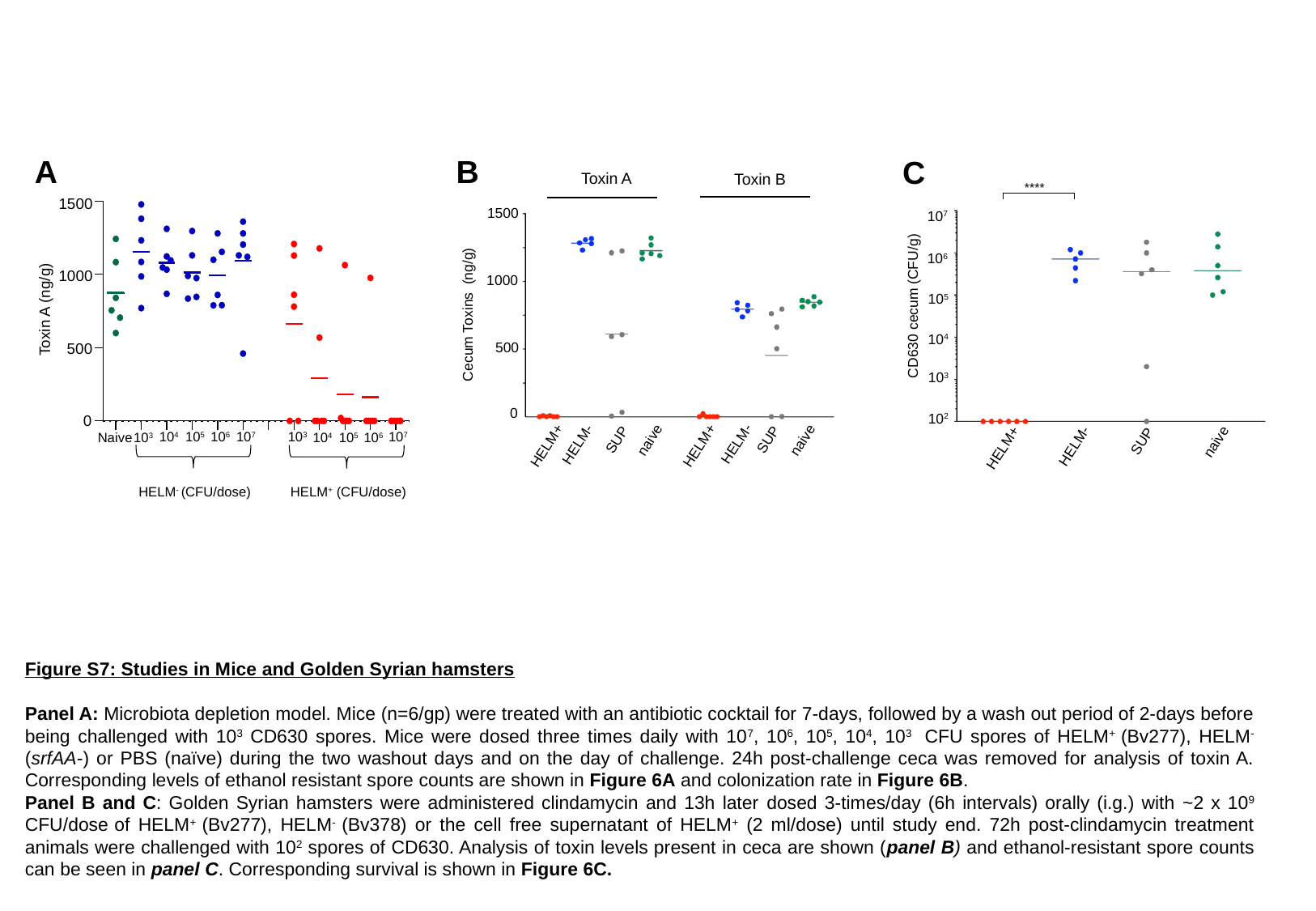

B
A
C
Toxin A
Toxin B
1500
1000
500
0
SUP
naive
SUP
naive
HELM-
HELM-
HELM+
HELM+
1500
1000
Toxin A (ng/g)
500
0
103
107
104
105
106
107
103
104
105
106
Naive
HELM- (CFU/dose)
HELM+ (CFU/dose)
****
107
106
105
CD630 cecum (CFU/g)
Cecum Toxins (ng/g)
104
103
102
SUP
naive
HELM-
HELM+
Figure S7: Studies in Mice and Golden Syrian hamsters
Panel A: Microbiota depletion model. Mice (n=6/gp) were treated with an antibiotic cocktail for 7-days, followed by a wash out period of 2-days before being challenged with 103 CD630 spores. Mice were dosed three times daily with 107, 106, 105, 104, 103 CFU spores of HELM+ (Bv277), HELM- (srfAA-) or PBS (naïve) during the two washout days and on the day of challenge. 24h post-challenge ceca was removed for analysis of toxin A. Corresponding levels of ethanol resistant spore counts are shown in Figure 6A and colonization rate in Figure 6B.
Panel B and C: Golden Syrian hamsters were administered clindamycin and 13h later dosed 3-times/day (6h intervals) orally (i.g.) with ~2 x 10^9^ CFU/dose of HELM+ (Bv277), HELM- (Bv378) or the cell free supernatant of HELM+ (2 ml/dose) until study end. 72h post-clindamycin treatment animals were challenged with 102 spores of CD630. Analysis of toxin levels present in ceca are shown (panel B) and ethanol-resistant spore counts can be seen in panel C. Corresponding survival is shown in Figure 6C.

## Slide 8
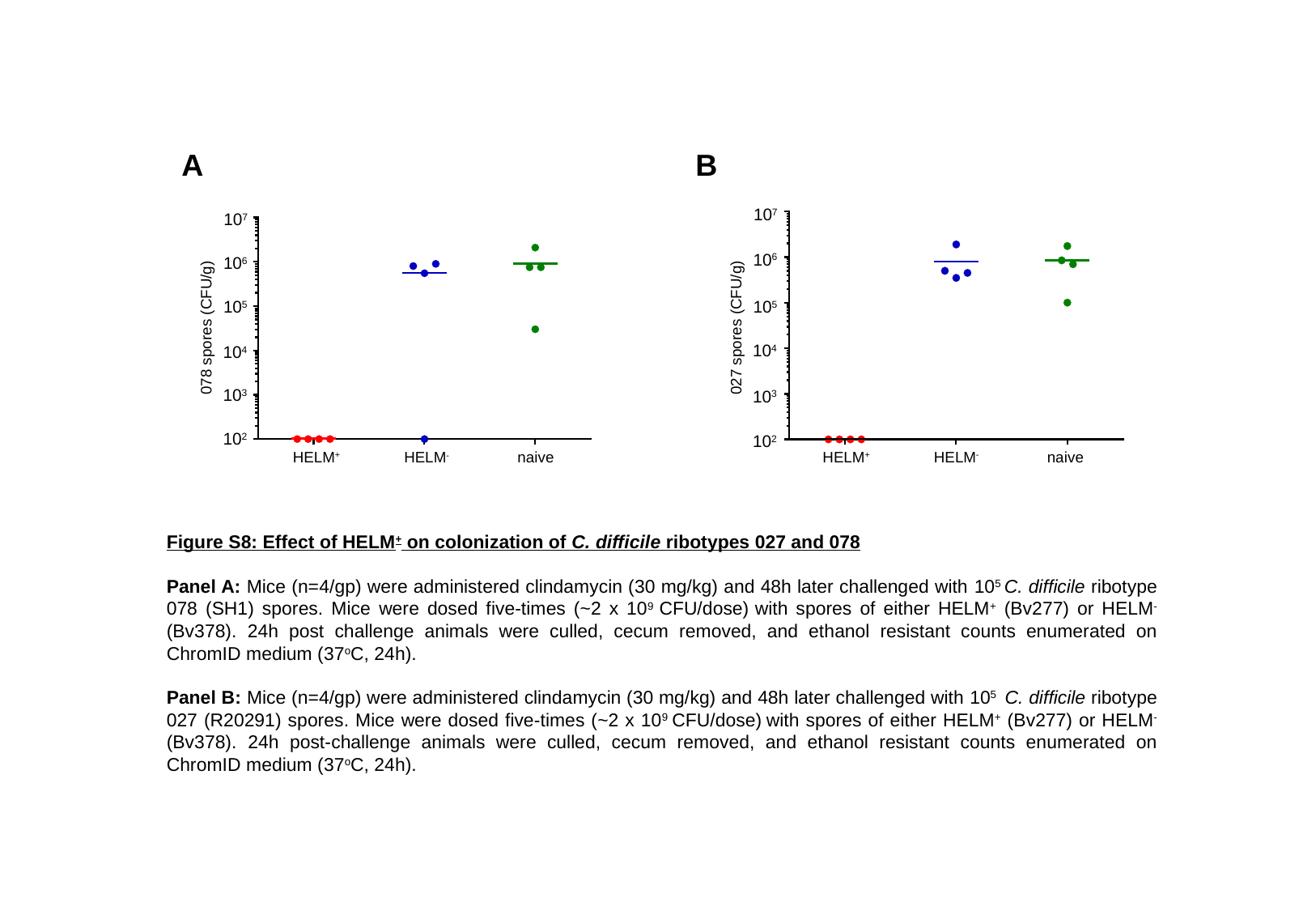

A
B
107
106
105
027 spores (CFU/g)
104
103
102
HELM+
naive
HELM-
107
106
105
078 spores (CFU/g)
104
103
102
HELM+
naive
HELM-
Figure S8: Effect of HELM+ on colonization of C. difficile ribotypes 027 and 078
Panel A: Mice (n=4/gp) were administered clindamycin (30 mg/kg) and 48h later challenged with 105 C. difficile ribotype 078 (SH1) spores. Mice were dosed five-times (~2 x 10^9^ CFU/dose) with spores of either HELM+ (Bv277) or HELM- (Bv378). 24h post challenge animals were culled, cecum removed, and ethanol resistant counts enumerated on ChromID medium (37oC, 24h).
Panel B: Mice (n=4/gp) were administered clindamycin (30 mg/kg) and 48h later challenged with 105 C. difficile ribotype 027 (R20291) spores. Mice were dosed five-times (~2 x 10^9^ CFU/dose) with spores of either HELM+ (Bv277) or HELM- (Bv378). 24h post-challenge animals were culled, cecum removed, and ethanol resistant counts enumerated on ChromID medium (37oC, 24h).
