# Supplementary material for: Environmentally Acquired Bacillus and Their Role in C. difficile Colonization Resistance
Source: Biomedicines. 2022 Apr 19;10(5):930. doi: 10.3390/biomedicines10050930 (PMC9138776; doi:10.3390/biomedicines10050930)
Supplement: Supplementary file 1 [file biomedicines-10-00930-s001.zip › Supplementary Methods.pdf]

Supplementary Methods

Strains

CD630 (or 630) was isolated from a patient with pseudomembranous colitis during an outbreak of *C. difficile* infection (CDI)[1]. The strain is ribotype 012 and produces toxins A (*tcdA*$^+$) and B (*tcdB*$^+$). Ribotype 078 (SH11) and 027 (R2091) strains were obtained from Public Health England or B. Wren respectively.

Construction of *srfAA* mutant

The genome of Bv277 (HELM$^+$) was sequenced in its entirety revealing the four cistrons of the *srfA* operon. Primers (F: AAAAAGCTTCGGGGTTCGGGAAACTCATTTC; R: TTTGAATTCGCATGCGCAGGGGTTCCGTCAG) were used to amplify a 2007 bp internal segment of the *srfAA* cistron of Bv277. Restriction endonuclease sites incorporated in the PCR primers enabled direct cloning of the amplified DNA into pSGMU2, a plasmid able to replicate autonomously in *E.coli* but not *Bacillus*[2]. Protoplasts of Bv277 cells were made using lysozyme degradation in an isotonic solution as described elsewhere[3] and DNA of the recombinant plasmid introduced followed by two-step phased selection of chloramphenicol resistance (5 µg ml^{-1}). Transformants would only arise if plasmid DNA had integrated at the *srfAA* cistron by virtue of homologous DNA between genome and plasmid. Integration by a Campbell-type recombination disrupts the *srfA* operon resulting in failure to transcribe all four cistrons.

Analysis of toxins using ELISA

Toxins A and B were recovered from fecal or cecal samples and diluted 1:5 (w/v) in extraction buffer (PBS containing 2% (v/v) fetal calf serum, penicillin-streptomycin (Sigma P4333; 10ml/L) and Pierce protease inhibitor tablets (Thermo 88265). Samples were homogenized in extraction buffer and incubated for 2h at 4°C. The supernatant was harvested after centrifugation (12,000 g, 5 min.) and filtered (0.2 µm). Rabbit anti-toxin A and toxin B (in house reagents) were used to coat ELISA plates (1/6,000) and left overnight at RT. Subsequently, plates were blocked for 1h at 37°C with 2% BSA. Toxin extracted samples were incubated for 2h at RT. Replicate samples were used together with a negative control (toxin extract from a non-infected animal). Detection antibodies were mouse anti-toxin A and anti-toxin B (in house reagents; 1/1000) and were incubated for 1h at 30°C. HRP-conjugated anti-mouse IgG (Dako; 1/2000) was used as the detection antibody for 1h at RT. Reactions were developed using TMB substrate and stopped by 2M H2SO4 and OD read at 450nm.

References

1. Wust, J., Sullivan, N. M., Hardegger, U. & Wilkins, T. D. Investigation of an outbreak of antibiotic-associated colitis by various typing methods. *J Clin Microbiol* **16**, 1096-1101, doi:10.1128/JCM.16.6.1096-1101.1982 (1982).

2. Fort, P. & Errington, J. Nucleotide sequence and complementation analysis of a polycistronic sporulation operon, *spoVA*, in *Bacillus subtilis*. *J Gen Microbiol* **131**, 1091-1105, doi:10.1099/00221287-131-5-1091 (1985).

3, Vehmaanpera, J. Transformation of *Bacillus amyloliquefaciens* protoplasts with plasmid DNA. *FEMS Microbiology Letters* **49**, 101-105 (1988).
