# Supplementary material for: Environmentally Acquired Bacillus and Their Role in C. difficile Colonization Resistance
Source: Biomedicines. 2022 Apr 19;10(5):930. doi: 10.3390/biomedicines10050930 (PMC9138776; doi:10.3390/biomedicines10050930)
Supplement: Supplementary file 1 [file biomedicines-10-00930-s001.zip › Supplementary Tables.pdf]

Supplementary Tables

Supplementary Table S1: Human *Bacillus* Strains with Inhibitory Activity to *C. difficile*

Name[a]	Surf[b]	Activity[c]			HELM Composition[d]			
		Sup.	$AmSO_4$	Lytic	Iturin	Fengycin	Surfactin	Chlorotetaine
Bv277	+++	1:160	1:1280	+	+	+	+	+
Bv297	+++	1:160	1:1280	+	+	+	+	+
Bv57	++	1:80	1:640	+	+	+	+	-
Bv137	++	1:80	1:640	+	-	+	+	-
Bv185	++	1:80	1:320	+	-	+	+	-
Bs83	+	1:20	1:320	+	-	+	+	-
Bs140	+	1:10	1:80	+	-	+	+	-

[a] Strains isolated from human feces were either *B. subtilis* (Bs) or *B. velezensis* (Bv). Species determination was made initially by sequencing of the *gyrA* gene and then by complete genome sequencing.

[b] Biosurfactant activity of cell free supernatants determined using an oil-displacement assay (Youssef, N. H. *et al.* Comparison of methods to detect biosurfactant production by diverse microorganisms. *J Microbiol Methods* **56**, 339-347, doi:10.1016/j.mimet.2003.11.001 (2004)).

[c] Activity was determined using an *in vitro* microdilution assay measuring inhibition of growth of CD630 using two test samples, a) a filter-sterile cell-free supernatant (Sup.) from an overnight (18h) culture of the *Bacillus* strain, and b) an $AmSO_4$ (20%) precipitate of the cell-free supernatant at approx. 30X concentration. Lytic activity was determined as the ability to clarify a log-phase culture of CD630.

[d] The $AmSO_4$ precipitate was fractionated using SEC and the active fraction (determined using the *in vitro* microdilution assay) examined by RP-HPLC. Approximately 20 fractions were collected and identified by cross-referencing them to the known retention time of each of the four compounds shown in the table.

Supplementary Table S2: MALDI-TOF Analysis of BV277 HELM RP-HPLC Active Fractions

Compounds	m/z	Ions detected	Structure
**Chlorotetaine**			
Chlorotetaine (^{35}Cl)	288.088	[M+Na], [M+K], [M-H+2xNa]	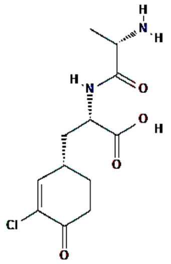
Chlorotetaine (^{37}Cl)	290.085	[M+Na], [M+K], [M-H+2xNa]	
Hydroxychlorotetaine (^{35}Cl)	304.083	[M+Na], [M+K]	
Hydroxychlorotetaine (^{37}Cl)	306.080	[M+Na], [M+K]	
**Iturins**			
C_{12}IturinA/C_{12}Mycosubtilin/C_{11}BacillomycinF	1014.512	[M+Na]	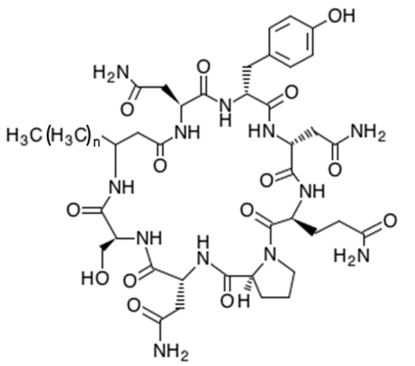
C_{14}BacillomycinL	1020.511	[M+Na], [M-H+2xNa]	
C_{13}IturinA/C_{13}Mycosubtilin/C_{12}BacillomycinF	1028.527	[M+Na]	
C_{14}BacillomycinD	1030.532	[M+K]	
C_{15}BacillomycinL	1034.527	[M+H], [M+Na], [M+K]	
C_{14}IturinA/C_{14}Mycosubtilin/C_{13}BacillomycinF	1042.543	[M+H], [M+Na], [M+K]	
C_{15}BacillomycinD	1044.547	[M+K]	
C_{16}BacillomycinL	1048.542	[M+H], [M-H+2xNa]	
C_{15}IturinA/C_{15}Mycosubtilin/C_{14}BacillomycinF	1056.559	[M+H], [M+Na], [M+K]	
C_{15}IturinB	1057.543	[M+H], [M+Na], [M+K]	
C_{16}BacillomycinD	1058.563	[M+K]	
C_{17}BacillomycinL	1062.558	[M-H+2xNa]	
C_{16}IturinA/C_{16}Mycosubtilin/C_{15}BacillomycinF	1070.574	[M+Na], [M+K]	
C_{17}IturinA/C_{17}Mycosubtilin/C_{16}BacillomycinF	1084.605	[M+Na]	

**Fengycins**			
C_{15}FengycinA/C_{13}FengycinB//Plipastatin B C_{13}	1448.778	[M+Na]	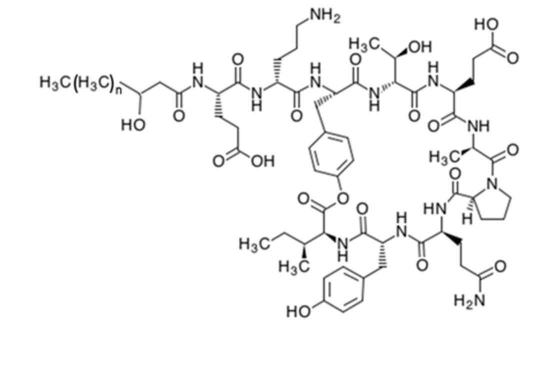
C_{16}FengycinA/C_{14}FengycinB//Plipastatin A1	1462.793	[M+H], [M+Na]	
Fengycin B (C_{16}/Ala-6)	1462.900	[M+H], [M+Na]	
C_{17}FengycinA/C_{15}FengycinB//Plipastatin A 2	1476.809	[M+H], [M+Na]	
Fengycin C (C_{17}/Ala-6)	1476.900	[M+H], [M+Na]	
C_{18}FengycinA/C_{16}FengycinB//Plipastatin B 1	1490.824	[M+H], [M+Na]	
Fengycin D (C_{16}/Val-6)	1490.900	[M+H], [M+Na]	
C_{19}FengycinA/C_{17}FengycinB//Plipastatin B 2	1504.840	[M+Na]	
Fengycin E (C_{17}/Val-6)	1504.900	[M+Na]	
**Surfactins**			
C_{12}SurfactinA/C_{13}SurfactinB/C_{12}SurfactinC/Pumilacidin	993.634	[M+Na], [M-H+Na+K]	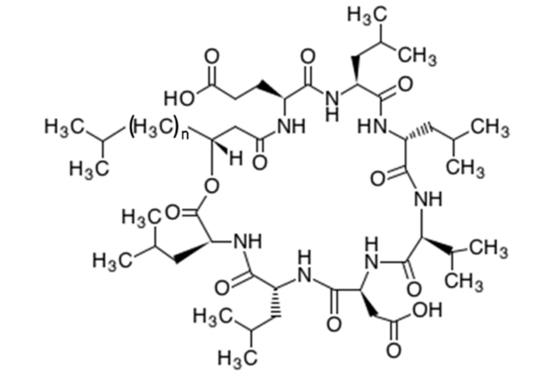
C_{13}SurfactinA/C_{14}SurfactinB/C_{13}SurfactinC/Pumilacidin	1007.650	[M+Na], [M+K], [M-H+2xNa], [M-H+Na+K]	
C_{12} Surfactin (linearized)	1011.600	[M+Na], [M-H+2xNa]	
C_{14}SurfactinA/C_{15}SurfactinB/C_{14}SurfactinC/Pumilacidin	1021.665	[M+Na], [M+K], [M-H+2xNa], [M-H+Na+K]	
C_{13} Surfactin (linearized)	1026.600	[M+K]	
C_{15}SurfactinA/C_{16}SurfactinB/C_{15}SurfactinC/Pumilacidin	1035.681	[M+Na], [M+K], [M-H+2xNa], [M-H+Na+K]	
C_{14} Surfactin (linearized)	1040.600	[M+K]	
C_{16}SurfactinA/C_{17}SurfactinB/C_{16}SurfactinC/Pumilacidin	1049.696	[M+H], [M+Na], [M+K]	
C_{17}SurfactinA/C_{18}SurfactinB/C_{17}SurfactinC/Pumilacidin	1063.712	[M+K]	
