# Supplementary material for: Environmentally Acquired Bacillus and Their Role in C. difficile Colonization Resistance
Source: Biomedicines. 2022 Apr 19;10(5):930. doi: 10.3390/biomedicines10050930 (PMC9138776; doi:10.3390/biomedicines10050930)
Supplement: Supplementary file 1 [file biomedicines-10-00930-s001.zip › Supplementary Video.pdf]

Supplementary Video

A filter-sterile supernatant of a Bv277 culture (grown for 18h in BHIB at 37°C) was added to a late-log culture (OD600 = ~1) of CD630 at a ratio of 1:10 (left tube). Incubation of CD630 was maintained at 37°C. After 28 minutes the treated culture showed almost complete lysis as compared to an untreated culture (right tube).
